# The impact of multifactorial stress combination on plant growth and survival

**DOI:** 10.1111/nph.17232

**Published:** 2021-02-18

**Authors:** Sara I. Zandalinas, Soham Sengupta, Felix B. Fritschi, Rajeev K. Azad, Rachel Nechushtai, Ron Mittler

**Affiliations:** ^1^ Division of Plant Sciences College of Agriculture Food and Natural Resources and Interdisciplinary Plant Group Christopher S. Bond Life Sciences Center University of Missouri 1201 Rollins St Columbia MO 65211 USA; ^2^ Department of Biological Sciences and BioDiscovery Institute College of Science University of North Texas 1155 Union Circle #305220 Denton TX 76203‐5017 USA; ^3^ Department of Mathematics University of North Texas Denton TX 76203 USA; ^4^ The Alexander Silberman Institute of Life Science The Hebrew University of Jerusalem Edmond J. Safra Campus at Givat Ram Jerusalem 91904 Israel; ^5^ Department of Surgery University of Missouri School of Medicine Christopher S. Bond Life Sciences Center University of Missouri 1201 Rollins St Columbia MO 65211 USA

**Keywords:** abiotic stress, *Arabidopsis thaliana*, climate change, global warming, multifactorial stress combination, reactive oxygen species (ROS), stress combination, transcriptomics

## Abstract

- Climate change‐driven extreme weather events, combined with increasing temperatures, harsh soil conditions, low water availability and quality, and the introduction of many man‐made pollutants, pose a unique challenge to plants. Although our knowledge of the response of plants to each of these individual conditions is vast, we know very little about how a combination of many of these factors, occurring simultaneously, that is multifactorial stress combination, impacts plants.
- Seedlings of wild‐type and different mutants of *Arabidopsis thaliana* plants were subjected to a multifactorial stress combination of six different stresses, each applied at a low level, and their survival, physiological and molecular responses determined.
- Our findings reveal that, while each of the different stresses, applied individually, had a negligible effect on plant growth and survival, the accumulated impact of multifactorial stress combination on plants was detrimental. We further show that the response of plants to multifactorial stress combination is unique and that specific pathways and processes play a critical role in the acclimation of plants to multifactorial stress combination.
- Taken together our findings reveal that further polluting our environment could result in higher complexities of multifactorial stress combinations that in turn could drive a critical decline in plant growth and survival.

## Introduction

The accumulated impact of human life on our planet over the past several decades has resulted in the introduction of many extreme environmental conditions into our ecosystems and agricultural lands (e.g. Sala *et al*., 2000; Grimm *et al*., 2008; Lehmann & Rillig, 2014; Teuling, 2018; Rillig *et al*., 2019). These include climate change‐driven extreme and fluctuating weather events (e.g. heat waves, cold snaps, flooding, and/or prolonged drought), combined with harsh soil conditions (e.g. saline, basic, and/or acidic), different man‐made contaminants (e.g. heavy metals, microplastics, pesticides, antibiotics and persistent organic pollutants), radiation (e.g. UV), limited nutrient availability, and high content of airborne molecules and gases (e.g. ozone, burn particles, CO_2_). In addition to directly impacting plant growth and reproduction within many eco‐ and agricultural systems (e.g. Mittler & Blumwald, 2010; Lobell & Gourdji, 2012; Bailey‐Serres *et al*., 2019; Borghi *et al*., 2019), some of these environmental conditions were also shown to increase the vulnerability of plants to attack by different pathogens or insects (e.g. Desaint *et al*., 2020; Hamann *et al*., 2020; Cohen & Leach, 2020; Savary & Willocquet, 2020).

Although our knowledge of the response of plants to each of the above‐mentioned extreme environmental conditions is vast, we know very little about how a combination of many of these factors, occurring simultaneously, that is multifactorial stress combination, would impact plant growth, reproduction, interactions with other organisms, and/or overall survival, and shape our future. It was recently demonstrated for example that increasing the number and complexity of different co‐occurring environmental stress factors, associated with global climatic changes, resulted in a gradual decline in soil properties, processes, and microbial populations (Rillig *et al*., 2019). Nevertheless, our understanding of how complex environmental conditions, occurring during a multifactorial stress combination, impact plant growth and survival is at best rudimentary.

Simple stress combination experiments (i.e. a combination of two or at maximum three different stresses), revealed that the response of plants to conditions of stress combination is unique and cannot be predicted by studying the response of plants to each of the different single stresses that compose the stress combination, applied individually (e.g. Rizhsky *et al*., 2004; Mittler, 2006; Mittler & Blumwald, 2010; Prasch & Sonnewald, 2013; Suzuki *et al*., 2014; Choudhury *et al*., 2017; Shaar‐Moshe *et al*., 2017, 2019; Zhang & Sonnewald, 2017; Balfagón *et al*., 2019; Zandalinas *et al*., 2020a). Plants display therefore a complex and plastic response to stress combination that may include components of the response to each of the individual stresses that compose the stress combination, as well as a large array of different transcripts, metabolites and proteins unique to the stress combination.

Exploring some of the mechanisms utilised by different prokaryotic organisms to withstand extreme and complex environmental conditions, highlights pathways and proteins that regulate the levels of reactive oxygen species (ROS) and iron in cells, as well as participate in protein and/or DNA repair and recycling, as essential for survival (e.g. Slade & Radman, 2011; Yuan *et al*., 2012; Mittler, 2017; Shuryak, 2019). It is possible therefore that acclimation to multifactorial stress conditions in plants would require similar mechanisms, and that these could be regulated by different and perhaps unique multifactorial stress‐specific transcriptomic networks.

To begin addressing the response of plants to multifactorial stress combination, we subjected seedlings of Arabidopsis plants grown in peat soil or on plates to a combination of six representative abiotic stress conditions (heat, salt, excess light, acidity, heavy metal, and oxidative stress imposed by the herbicide paraquat) and studied their growth, survival and molecular responses.

## Materials and Methods

### Plant material and stress treatments

Seeds of *Arabidopsis thaliana* wild‐type Col‐0, respiratory burst oxidase homologue D (*rbohD*; Fichman *et al*., 2019), cytosolic ascorbate peroxidase 1 (*apx1*; Davletova *et al*., 2005), allene oxide synthase (*aos*; Balfagón *et al*., 2019), salicylic acid‐induction deficient 2 (*sid2*; Nawrath & Métraux, 1999), ethylene‐insensitive protein 2 (*ein2*; Alonso *et al*., 1999), abscisic acid (ABA) deficient 2 (*aba2*; González‐Guzmán *et al*., 2002), multiprotein bridging factor 1c (*mbf1c*; Suzuki *et al*., 2011), autophagy‐related protein 9 (*atg9*; Floyd *et al*., 2015), and AtNEET‐overexpressing and RNAi plants (Nechushtai *et al*., 2012; Zandalinas *et al*., 2020b) were sterilised and placed on rectangular (12 cm width) 1% agar vertical plates containing ½ Murashige and Skoog (½MS) medium at pH 5.8. Next, 25–30 seeds of each genotype were placed side‐by‐side on the same plate, and each treatment was repeated using three biological replicates for a total of 75–90 seeds per treatment, per genotype (Luhua *et al*., 2008, 2013). Sterilised seeds of the different genotypes were then subjected to the following individual treatments and their different combinations: CT (control, ½MS, 21°C, 50 µmol m^−2^ s^−1^, pH 5.8), Acidity (½MS, 21°C, 50 µmol m^−2^ s^−1^, buffered to pH 5.0), Cd (½MS, 21°C, 50 µmol m^−2^ s^−1^, pH 5.8, 5 µM CdCl_2_), HL (high light, ½MS, 21°C, pH 5.8, 700 µmol m^−2^ s^−1^), HS (heat stress, ½MS, 50 µmol m^−2^ s^−1^, pH 5.8, 33°C), Salt (½MS, 21°C, 50 µmol m^−2^ s^−1^, pH 5.8, 50 mM NaCl), and PQ (½MS, 21°C, 50 µmol m^−2^ s^−1^, pH 5.8, 0.05 µM paraquat) (Luhua *et al*., 2008, 2013; Zandalinas *et al*., 2020b). For stress combinations involving HL and/or HS, seeds were allowed to germinate and grow in the presence or absence of the other stress conditions (CT, Acidity, Cd, Salt and/or PQ) for 6 d and then subjected to a 3‐d treatment of HL and/or HS. For abiotic stresses and their combinations not involving HL and/or HS, seeds were allowed to germinate and grow in the presence or absence of stress conditions for 9 d. Percent survival and root length were measured for all plates at the same time (9 d), followed by sampling of seedlings for chlorophyll extraction as described (Luhua *et al*., 2008, 2013; Zandalinas *et al*., 2020b; using five biological repeats). Seedlings grown on separate sets of horizontal plates were subjected to the different individual or combined stresses as described above, but sampled together 1.5 h (for RNA‐Seq analysis), or 3 h (for imaging ROS; Fichman *et al*., 2019), in three biological repeats, following the initiation of the HS and/or HL stresses. For experiments of multifactorial stress combination in peat soil, Col, *apx1* and *rbohD* seeds were germinated and grown in peat pellets (Jiffy‐7, Jiffy; http://www.jiffygroup.com/) at 21°C and 50 µmol m^−2^ s^−1^, and watered periodically with the following solutions and their different combinations: CT (water; pH 7.2), Salt (50 mM NaCl), PQ (0.05 µM paraquat), acidity (water; buffered to pH 5 with HCl), and Cd (5 µM CdCl_2_). At 7 d following germination, seedlings grown under the different conditions described above were untreated further or subjected to heat stress (HS; 33°C, 50 µmol m^−2^ s^−1^), and/or high light stress (HL; 21°C, 700 µmol m^−2^ s^−1^), for 3 d. All peat soil‐grown seedlings were sampled 10 d following germination, and percent survival, seedling diameter, ROS imaging and chlorophyll content were determined as described above (Luhua *et al*., 2008, 2013; Fichman *et al*., 2019; Zandalinas *et al*., 2020b).

To study multifactorial stress combination in Arabidopsis, HS, HL, Salt and PQ stresses were conducted in all possible combinations (Salt, PQ, HL, HS, Salt + PQ, Salt + HL, Salt + HS, PQ + HL, PQ + HS, HL + HS, Salt + PQ + HL, Salt + PQ + HS, Salt + HL + HS, PQ + HL + HS, Salt + PQ + HL + HS), and acidity and Cd were added to our analysis as single stresses, as well as in combination with Salt + PQ + HL + HS to generate two different five‐stress (Salt + PQ + HL + HS + Acidity, and Salt + PQ + HL + HS + Cd) and one six‐stress (Salt + PQ + HL + HS + Acidity + Cd) combination states. As a result, single and all multifactorial combinations could be studied for HS, HL, Salt and PQ stresses, but not for all combinations that included Cd and acidity. For each treatment conducted, we used a minimum of *n* = 75 replication level for root and rosette growth and ROS accumulation analyses, *n* = 5 for chlorophyll determination, and *n* = 3 for RNA‐Seq and ROS analyses (please see below). The impact of Cd and/or acidity could therefore be studied only as added stresses, while the impact of HS, HL, Salt and PQ stresses could be studied in all combinations. This means that, when it comes to Cd and acidity, our resolution does not allow statements on all specific or individual factor interactions involving these two stressors. Addressing all possible interactions for the six different stresses would have resulted in an experimental design encompassing all factor combinations with 64 unique treatments per each of the three genotypes (a total of 192), which, applying our level of replication, would mean at least 14 400 experimental units. An approach similar to the one described in this study was used by Rillig *et al*., (2019) to study the impact of multifactorial stress combination on soil properties, processes and microbial populations.

### RNA sequencing (RNA‐Seq) and analysis

At least 100, 9 to 10‐d‐old Col‐0 seedlings, growing on 1% horizontal plates were subjected to the different control and stress combination treatments as described above in three biological replicates. For RNA‐Seq experiments, HS, HL, Salt and PQ were conducted in all possible combinations and acidity and Cd were added to the four‐stress combination state to generate two different five‐stress and one six‐stress combination states, as described above. Total RNA was isolated and subjected to RNA‐Seq analysis as described in Zandalinas *et al*. (2019, 2020b). Briefly, single‐end sequenced reads were quality tested using fastqc v.0.11.7 (Andrews, 2010) and aligned to the reference genome of Arabidopsis (genome build 10) obtained from TAIR (https://www.arabidopsis.org/) using Star aligner v.2.4.0.1 (Dobin *et al*., 2013). Default mapping parameters (10 mismatches/read; nine multi‐mapping locations/read) were used. The genome index was generated using the gene annotation file (GFF) obtained from TAIR (Araport11; https://www.arabidopsis.org/download_files/Genes/TAIR10_genome_release/TAIR10_gff3/TAIR10_GFF3_genes.gff) for the genome build 10. Differential gene expression analysis was carried out using deseq2, an R based package available from Bioconductor (Love *et al*., 2014), with mapped read counts generated using Star aligner v.2.4.0.1 (Dobin *et al*., 2013). Genes differentially expressed in two (or more) conditions were identified by comparing mapped read abundance under the different conditions. Gene expression was measured as mean normalised counts of reads mapped onto the different genes (Love *et al*., 2014). The difference in expression was quantified in terms of the logarithm (log_2_) of the ratio of mean normalised counts between two conditions (log fold change). Differentially expressed genes were defined as those that have a log fold change with an FDR‐adjusted *P*‐value < 0.05 (negative binomial Wald test followed by Benjamini–Hochberg correction; Love *et al*., 2014). Genes with zero raw fold‐change expression value were omitted from further analysis. Differentially expressed genes were classified into upregulated or downregulated based on significant positive or negative log fold‐change values, respectively. Venn diagram overlap was calculated using (http://bioinformatics.psb.ugent.be/webtools/Venn/). Functional annotation and quantification of overrepresented GO terms were conducted using David 6.8, heat maps were generated using mev v.4.9.0 software, and Venn diagram overlaps were subjected to statistical significance tests (based on hypergeometric distribution) using phyper (R package) (Zandalinas *et al*., 2019, 2020b). Perl scripts used in this study were uploaded to: https://github.com/sohamsg90/RNA‐Seq‐perl‐scripts. RNA‐Seq analyses results are shown in Supporting Information Tables S1–S49.

### ROS detection and measurement

ROS imaging was conducted using 25–30, 9–10‐d‐old seedlings, of the different genotypes, subjected to the different stress combinations while growing on plates or in peat soil as described (Fichman *et al*., 2019), using three biological repeats. ROS accumulation was analysed using Living Image v.4.7.2 software (Perkin Elmer) utilising the math tools. Images were generated and total radiant efficiency (TRE) of regions of interest (ROI) were calculated as described (Fichman *et al*., 2019). Radiant efficiency is defined as fluorescence emission radiance per incident excitation and is expressed as (p/s)/(µW cm^−2^); p, photons; s, seconds; μW, microWatt; cm^2^, square centimetre (Fichman *et al*., 2019).

### Statistical analysis

All experiments were repeated at least three times. Results are presented as the mean ± SD. Statistical analyses were performed using two‐way ANOVA followed using a Tukey *post hoc* test (different letters/asterisks denote statistical significance at *P* < 0.05; Interaction terms and their associated *P*‐values are shown in Table S50). Statistical significance of Venn diagram overlap was determined by performing a statistical hypothesis test based on hypergeometric distribution using the R package phyper (http://nemates.org/MA/progs/overlap_stats.html; Zandalinas *et al*., 2019, 2020b).

### Accession numbers

All data are available in the main text or the Supporting Information files. RNA‐Seq data files were deposited in Gene Expression Omnibus (GEO) (https://www.ncbi.nlm.nih.gov/geo/) under the accession no. GSE147962.

## Results

### Survival and growth of Arabidopsis seedlings subjected to multifactorial stress combination

**Fig. 1:**
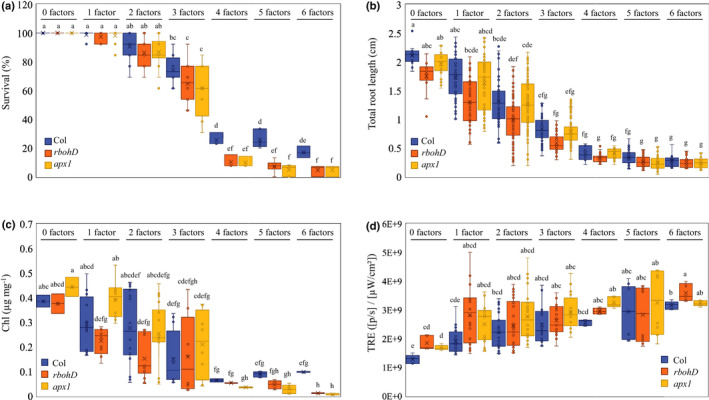
The impact of multifactorial stress combinations on growth and survival of *Arabidopsis thaliana* seedlings. (a–d) The effect of multifactorial stress conditions (heat, salt, excess light, acidity, heavy metal, and oxidative stresses) applied in different combinations (up to a combination of all six factors) was determined on the survival (a), root growth (b), chlorophyll content (c) and whole‐plant ROS levels (d), of wild‐type, *rbohD* and *apx1* seedlings. Box plots show the median (horizontal line), the lower and upper bounds of each box plot denote the first and third quartiles (the 25^th^ and 75^th^ percentiles, respectively), and whiskers above and below the box plot indicate 1.5 times the interquartile range. Statistical analysis was performed by two‐way ANOVA followed by a Tukey post hoc test (different letters denote statistical significance at *P* < 0.05; Table S50). Abbreviations: Apx1, ascorbate peroxidase 1; Chl, chlorophyll; RbohD, respiratory burst oxidase homologue D; TRE, total radiant efficiency.

**Fig. 2:**
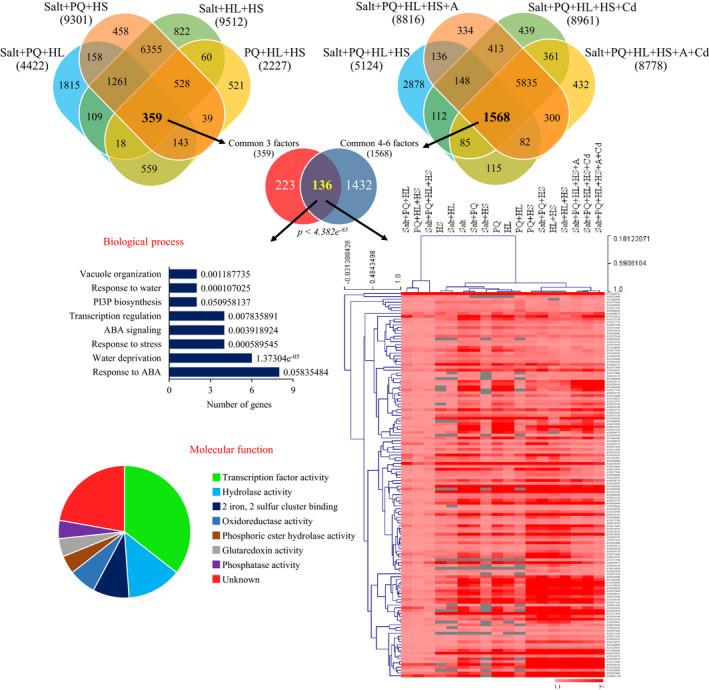
Gene expression analysis of multifactorial stress responses. Gene expression analysis of the response of *Arabidopsis thaliana* seedlings to different multifactorial stress combinations of heat, salt, excess light, oxidative stress (induced by the herbicide paraquat), acidity and heavy metal (cadmium) is shown (see also Fig. S5). Venn diagrams depicting the overlap between genes upregulated in their expression in response to several different three‐factor stress combinations (left), or four‐, five‐ and six‐stress factor combinations (right) are shown on top. A Venn diagram showing the overlap between genes upregulated in their expression in response to several different three‐factor stress combinations and genes upregulated in their expression in response to four‐, five‐ and six‐ stress factor combinations (136 genes) is shown underneath, together with bar and pie charts of biological process and molecular function (GO) annotations for these genes, and a heat map showing the expression level and clustering of these genes under all treatment combinations tested. Statistical significance of Venn diagram overlap was determined by hypergeometric testing analysis using the R package phyper (Table S50). Abbreviations: A, acidity; ABA, abscisic acid; Cd, cadmium; HL, high light; HS, heat stress; PI3P, phosphatidylinositol 3‐phosphate; PQ, paraquat.

**Fig. 3:**
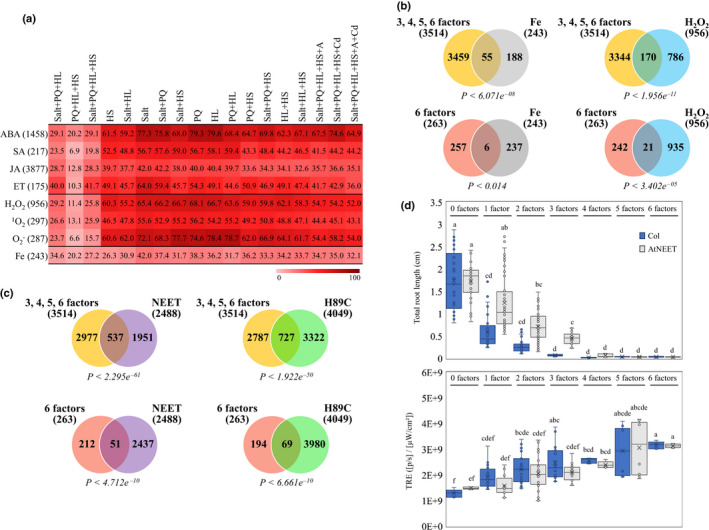
Enriched content of ROS‐, iron‐ and stress‐related genes in the response of plants to multifactorial stress combinations, and the impact of multifactorial stress combinations (heat, salt, excess light, acidity, heavy metal, and oxidative stresses) on root growth of *Arabidopsis thaliana* wild‐type and AtNEET‐overexpressing seedlings. (a) Heat map showing the representation (%) of hormone‐, ROS‐ and iron‐response genes among the different genes upregulated in their expression in response to multifactorial stress combination (*P*‐values for % representation are shown in Table S50). (b) Venn diagrams depicting the overlap between genes altered in their expression in seedlings in response to a combination of three, four, five, and six different stresses (top), or genes common in their expression in response to all six different stress combinations (bottom), and genes altered in their expression in plants in response to elevated levels of H_2_O_2_ or alterations in iron levels. (c) Venn diagrams depicting the overlap between genes altered in their expression in seedlings in response to a combination of three, four, five, and six different stresses (top), or genes common to all six different stress combinations (bottom), and genes altered in their expression in seedlings that overexpress AtNEET or the AtNEET variant H89C. Statistical significance for Venn diagrams overlap in (b) and (c) was determined by hypergeometric testing analysis using the R package phyper (Table S50). (d) The impact of multifactorial stress combinations on root growth and whole‐plant ROS levels of wild‐type and AtNEET‐overexpressing seedlings. Box plots show the median (horizontal line), the lower and upper bounds of each box plot denote the first and third quartiles (the 25^th^ and 75^th^ percentiles, respectively), and whiskers above and below the box plot indicate 1.5 times the interquartile range. Statistical analysis was performed by two‐way ANOVA followed by a Tukey post hoc test (different letters denote statistical significance at *P* < 0.05; Table S50). Abbreviations: A, acid; ABA, abscisic acid; ET, ethylene; HL, high light stress; HS, heat stress; JA, jasmonic acid; PQ, paraquat; SA, salicylic acid; ROS, reactive oxygen species; TRE, total radiant efficiency.

**Fig. 4:**
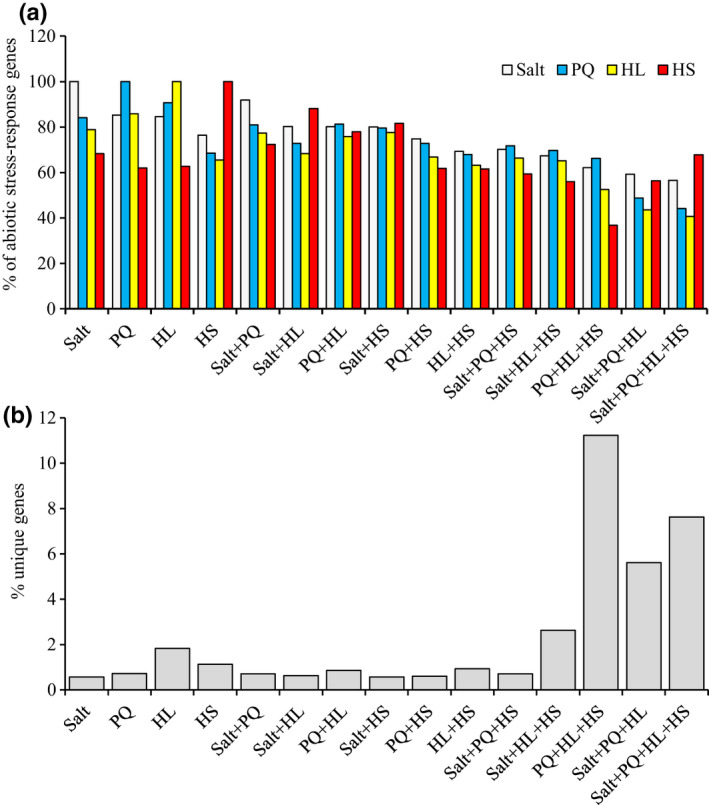
Representation of single‐stress differentially regulated genes shared by other single stresses under single or combinatorial stress conditions in *Arabidopsis thaliana* seedlings. (a) Representation of Salt, heat (HS), high light (HL)‐ and paraquat (PQ)‐induced genes in plants subjected to a multifactorial stress combination of Salt, HS, HL and PQ in all possible combinations. The % representation of the four different stress‐response gene groups (Salt, HS, HL and PQ) is shown for all possible combinations of the multifactorial stress combination (All are significant; *P*‐values are shown in Table S50). (b) Representation of unique gene expression patterns (as % of total number of genes significantly expressed in response to each treatment), in plants subjected to a multifactorial stress combination of Salt, HS, HL and PQ in all possible. Abbreviations: HL, high light; HS, heat stress; PQ, paraquat.

**Fig. 5:**
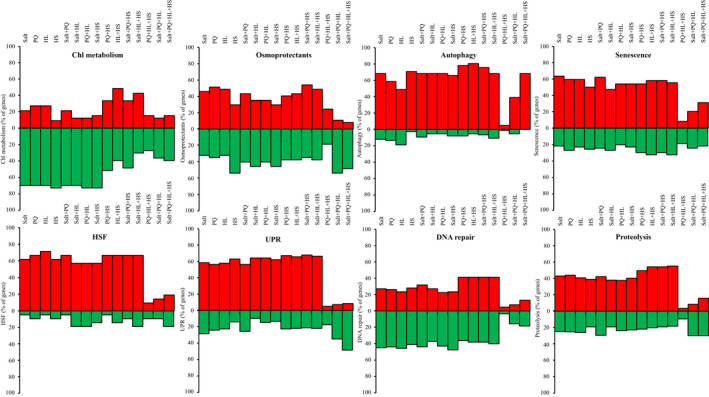
Expression of genes involved in chlorophyll and osmoregulation metabolism, autophagy, DNA repair, proteolysis, senescence and HSF and UPR pathways during the responses of *Arabidopsis thaliana* plants to multifactorial stress combinations. Percentage of gene expression changes, out of total genes associated with each of the different pathways (upregulated or downregulated; Tables S42–S49), is shown for all possible combinations of the salt, heat, high light and paraquat multifactorial stress combinations. Abbreviations: Chl, chlorophyll; HL, high light; HS, heat stress; HSF, heat shock factor; PQ, paraquat; UPR, unfolded protein response.

**Fig. 6:**
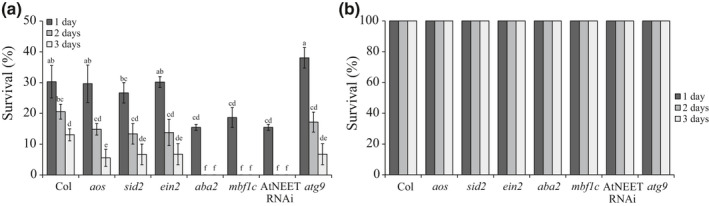
The impact of multifactorial stress combination on the survival of different *Arabidopsis thaliana* mutants. (a) The effect of a multifactorial stress combination of six different stresses (heat, salt, excess light, acidity, heavy metal and oxidative stresses) on the survival of wild‐type, *aos*, *sid2*, *ein2*, *aba2*, *mbf1c*, AtNEET RNAi and *atg9* seedlings. (b) Survival of wild‐type, *aos*, *sid2*, *ein2*, *aba2*, *mbf1c*, AtNEET RNAi and *atg9* seedlings under control conditions. Results are presented as the mean ± SD. Statistical analysis was performed using two‐way ANOVA followed using a Tukey post hoc test (different letters denote statistical significance at *P* < 0.05; Table S50). Abbreviations: *aba2*, abscisic acid deficient 2; *aos*, allene oxide synthase; *atg9*, autophagy‐related 9; *ein2*, ethylene‐insensitive 2; *mbf1c*, multiprotein bridging factor 1c; *sid2*, salicylic acid‐induction deficient 2.

*Arabidopsis thaliana* seedlings grown on agarose plates were subjected to a multifactorial stress combination of six different abiotic stress conditions including heat, salt, excess light, acidity, heavy metal, and oxidative stress (imposed by the herbicide paraquat), and their survival, root growth, chlorophyll and ROS levels determined (Figs 1, S1–S4). The rationale for using seedlings grown on plates, as opposed to soil, in our first set of experiments (Figs 1, 2, 3, 4, 5, 6, S1–S6), was to isolate and study the impact of multifactorial stress combination on plants in the absence of its impact on soils (Rillig *et al*., 2019). In addition, the use of plates enabled us to quantify root growth, a very sensitive measure of plant growth in the presence of stress (Luhua *et al*., 2008, 2013; Dubois & Inzé, 2020). To prevent lethality that could potentially result from conditions of stress combination, the intensities and duration of each of the individual stresses applied were calibrated based on our previous studies (Luhua *et al*., 2008, 2013), to ensure minimal impact on plant growth and survival (Figs 1, S1–S4). Markedly, while each of the individual stresses applied to seedlings had an overall minimal effect on plants, with the increasing number and complexity of multifactorial stress combinations, survival, root growth and chlorophyll content declined (Figs 1a–c, S1–S3). By contrast, an opposite trend was observed in whole‐plant ROS levels (Figs 1d, S4). These findings revealed that, although the effect of each individual stress on plant survival and growth is minimal (Figs 1, S1–S4), the accrued impact of multifactorial stress combination on plants is detrimental.

### Survival and growth of Arabidopsis seedlings under multifactorial stress conditions requires the function of two different genes involved in the regulation of ROS levels

Because ROS play a key role in the response of plants to almost all abiotic stresses studied to date (Van Breusegem *et al*., 2008; Choudhury *et al*., 2017; Mittler, 2017), we compared the multifactorial stress combination response of wild‐type seedlings to that of mutants impaired in ROS signalling (*rbohD*), or scavenging (*apx1*). As with wild‐type seedlings, survival, root growth and chlorophyll content of the *apx1* and *rbohD* seedlings declined with the increased number and complexity of multifactorial stress combinations (Figs 1a–c, S1–S3). However, compared with wild‐type, the decline in survival was overall augmented in the two mutants (Figs 1a, S1). In the absence of stress, the levels of ROS in the two mutants were higher compared with that of wild‐type, while in the presence of stress the overall levels of ROS were either similar or higher compared with wild‐type in the two mutants (Figs 1d, S4). These findings suggested that managing the overall levels of ROS in cells is essential for plant acclimation to multifactorial stress combination.

### Gene expression analysis of the response of Arabidopsis seedlings to multifactorial stress combination highlights unique and common gene expression patterns associated with multifactorial stresses

A gene expression RNA‐Seq study of Arabidopsis seedlings subjected to a representative set of multifactorial stress conditions that included six different stresses (i.e. Salt, PQ, HL, HS, Salt + PQ, Salt + HL, Salt + HS, PQ + HL, PQ + HS, HL + HS, Salt + PQ + HL, Salt + PQ + HS, Salt + HL + HS, PQ + HL + HS, Salt + PQ + HL + HS, Salt + PQ + HL + HS + Acidity, Salt + PQ + HL + HS + Cd, and Salt + PQ + HL + HS + Acidity + Cd) was conducted. As shown in Fig. S5a, each of the individual stresses analysed (i.e. Salt, PQ, HL, or HS) resulted in a gene expression response that contained genes unique to it, as well as genes shared with other stresses. Similarly, each of the different two‐stress combination states studied resulted in a gene expression response that contained unique and shared genes with other two‐stress combination states (Fig. S5b). Similar results were obtained for the higher‐level multifactorial combinations of three, four, five and six different stresses, cumulating in the identification of 8778 and 8766 genes as significantly enhanced or suppressed, respectively, in response to all six stresses combined (Figs 2, S5c,d; Tables S1–S41). As shown in Figs 2 and S5, in addition to gene expression patterns common between the different stresses and their different combinations, each different combination of stresses, defining a multifactorial stress condition, resulted in the expression of unique sets of genes induced only under its own set of multifactorial stress conditions (included within the representative set of multifactorial stress conditions studied). A set of 432 and 428 genes significantly enhanced or suppressed, respectively, was for example found to be unique to the state of six‐stress multifactorial combination (Figs 2, S5c; Tables S39–S41). These findings not only highlight the plasticity of the plant response to multifactorial stress combination, but also suggest that each different combination of stresses could result in a unique set of conditions that elicits a unique gene expression response. By contrast with the unique sets of gene expression patterns specific to each stress combination, the expression of 136 and 127 genes was significantly enhanced or suppressed, respectively, in response to all different multifactorial stress combinations included within the representative set of multifactorial stress conditions studied (Figs 2, S5c,d; Tables S37, S38). Interestingly, the expression pattern of some of these common genes clustered in a unique pattern suggesting that some multifactorial stress combinations (e.g. PQ + HL + HS, Salt + PQ + HL and Salt + PQ + HL + HS) are different compared with others (Fig. 2). The set of genes significantly enhanced in response to all multifactorial stress conditions studied included genes involved in the regulation of transcription, redox control, stress responses and the plant hormone ABA, as well as 2Fe–2S binding, hydrolase and glutaredoxin activities (Fig. 2). By contrast, the set of genes significantly suppressed in response to all multifactorial stress conditions included genes involved in amino acid and carbohydrate metabolism, heme‐binding, and glutathione transferase and peroxidase activities (Fig. S5c).

### The response of Arabidopsis to multifactorial stress combination involves genes encoding proteins associated with the regulation of iron and ROS levels in cells

Further analysis of gene expression during multifactorial stress combination revealed a high representation of ROS‐, iron‐ and other stress hormone‐response genes, such as ABA, jasmonic acid (JA), ethylene (ET), and salicylic acid (SA), among the genes with enhanced expression in all of the studied stress treatments, as well as their representative multifactorial combinations (Fig. 3a; RNA‐Seq data from the current study was compared with RNA‐Seq data from Zandalinas *et al*., 2019, 2020a,b). In addition, as shown in Fig. 3a,b considerable overlap was found between genes altered in their expression in seedlings in response to a combination of three, four, five, and six different stresses [359 + 1568 from Fig. 2, + 339 + 1511 from Fig. S5c = 3777, – (136 from Fig. 2, +127 Fig. S5c = 263) = 3514], or genes common in their expression in response to all six different stress combinations (136 from Fig. 2, + 127 from Fig. S5c = 263), and genes altered in their expression in plants in response to elevated levels of H_2_O_2_ or alterations in iron levels (from Zandalinas *et al*., 2020b). The different gene expression signatures described above (Figs 2, 3, S5), the established link between iron and ROS levels in different biological systems (Schieber & Chandel, 2014; Halliwell & Gutteridge, 2015; Mittler, 2017), and our findings that mutants impaired in ROS metabolism and signalling are highly sensitive to multifactorial stress combination (Figs 1a, S1), strongly suggest that managing iron and ROS levels could be crucial for plant survival under conditions of multifactorial stress combination.

Recent studies highlighted a key role for the iron–sulfur (2Fe–2S) protein AtNEET (At5g51720), and its mammalian counterparts (mitoNEET and NAF‐1), in the regulation of iron and ROS levels in cells (Nechushtai *et al*., 2012; Sohn *et al*., 2013; Darash‐Yahana *et al*., 2016; Mittler *et al*., 2019; Zandalinas *et al*., 2020b). While overexpression of the AtNEET protein had a negligible impact on Arabidopsis growth, a disruption in AtNEET function by overexpression of a dominant‐negative variant of AtNEET (H89C), resulted in the over‐accumulation of iron and ROS in cells and the premature death of seedlings (Zandalinas *et al*., 2020b). Interestingly, comparing the gene expression patterns of seedlings overexpressing AtNEET or H89C (from Zandalinas *et al*., 2020b) with that of seedlings subjected to a combination of three, four, five, and six different stresses (3514 genes; see above), or genes common in their expression in response to all six different stress combinations (263 genes; see above), revealed a significant overlap (Fig. 3c). Moreover, as shown in Figs 3d and S6, overexpressing AtNEET mitigated some of the effects of multifactorial stress combination on root growth. These findings are in agreement with the high representation of iron‐, ROS‐ and 2Fe‐2S‐related genes among the genes significantly enhanced in their expression in response to all six multifactorial stresses, and the overlap between gene expression patterns significantly altered in response to multifactorial stress combination and AtNEET‐ or H89C‐expressing plants (Figs 2, 3).

### Unique and common pathways associated with the response of plants to multifactorial stress combination

To further dissect the gene expression responses of Arabidopsis to multifactorial stress combinations, we focused on the complete set of Salt, PQ, HL and HS treatments and determined their relative gene expression patterns among all stresses and their combinations. As shown in Fig. 4a,b considerable overlap was found between the gene expression responses of Arabidopsis to each of the individual stress treatments (i.e. Salt, PQ, HL and HS), with 65–85% of genes included in each individual response showing a common response to the different single‐stress treatments (see also Fig. S5a). By contrast, once the different single stresses were combined (in two, three or four combinations), none of the gene expression responses to each individual stress reached its maximum and the degree of individual gene expression responses for each stress decreased as the combinations became more complex (Fig. 4a). By contrast with genes involved in the response of Arabidopsis to each individual stress (Fig. 4a), the percentage of unique gene expression responses associated with each different treatment or their combinations increased as the combinations became more complex, with the highest number of unique gene expression responses found for the combinations of PQ + HL + HS, Salt + PQ + HL and Salt + PQ + HL + HS (Fig. 4b). The findings presented in Fig. 4 suggest that with the increased complexity of stress combination, the number of genes responding to each individual stress decreases while the number of gene expression responses unique to the different stress combination(s) increases.

To determine the relative involvement of a representative set of different acclimation, defence and recycling pathways in the response of Arabidopsis to the different stresses and their combinations, we calculated the percentage of genes altered in their expression in different pathways (i.e. chlorophyll and osmoregulation metabolism, autophagy, DNA repair, proteolysis, senescence, and heat shock factor (HSF), and unfolded protein response (UPR) pathways; Tables S42–S49) in response to each individual stress and their combination. We chose this representative set of pathways based on prior studies suggesting that they could be important for plant acclimation to stress (Mittler & Blumwald, 2010; Zhu, 2016; Bailey‐Serres *et al*., 2019; Zandalinas *et al*., 2020a). As shown in Fig. 5, different stress combinations were different in the percentage of gene activation/suppression belonging to the different pathways. Of particular interest were stress combinations that included PQ + HL + HS, Salt + PQ + HL, and Salt + PQ + HL + HS. These combinations appeared to display a lower proportion of gene expression events associated with many of the pathways activated by the other stresses and their combinations (Fig. 5). Interestingly, the percentage of unique gene expression responses activated by these specific combinations was also higher compared with those found to be triggered by all other stresses and their combinations (Fig. 4b), suggesting that the response of Arabidopsis to these particular combinations (PQ + HL+HS, Salt + PQ + HL, and Salt + PQ + HL + HS) is different compared with that to many other stresses and their combinations and may involve pathways or metabolites with a defence/acclimation role, not identified/studied yet. Alternatively, during these combinations, Arabidopsis plants might enter a state of suppressed activity, or undergo cell death and therefore do not trigger many of the studied pathways. Direct evidence in support of the latter possibility was however not found in the survival, growth and chlorophyll content measurements conducted for these specific stress combinations (Figs S1–S4, S6). Further studies are of course needed to address these interesting possibilities.

To further study the involvement of different hormone‐response pathways (Fig. 3a), AtNEET (Fig. 3c,d), autophagy (Fig. 5), and thermotolerance (Fig. 5) in the acclimation of Arabidopsis plants to multifactorial stress combination, we compared the survival of mutants impaired in ABA (*aba2*; González‐Guzmán *et al*., 2002), JA (*aos*; Balfagón *et al*., 2019), SA (*sid2*; Nawrath & Métraux, 1999), ET (*ein2*; Alonso *et al*., 1999), AtNEET (AtNEET RNAi; Nechushtai *et al*., 2012), autophagy (*atg9*; Floyd *et al*., 2015) and basal thermotolerance (*mbf1c*; Suzuki *et al*., 2011) functions to a combination of six different stresses (Fig. 6). Interestingly, while the function of ABA2, MBF1c or AtNEET was absolutely required for plant survival under conditions of multifactorial stress combination of six different stresses, the function of AOS, SID2, EIN2 or ATG9 was not (Fig. 6). These findings support a role for ABA signalling, MBF1c‐regulated heat stress response, and AtNEET (Figs 3, 4, 5, 6), in the tolerance of Arabidopsis plants to multifactorial stresses.

### Survival and growth of Arabidopsis seedlings subjected to multifactorial stress combinations in peat soil

**Fig. 7:**
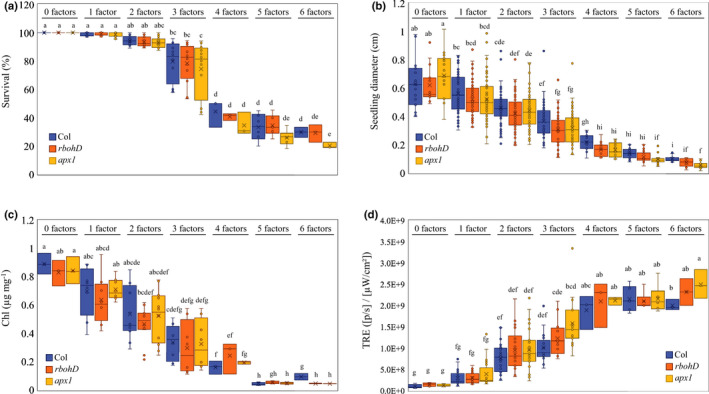
The impact of multifactorial stress combinations on growth and survival of *Arabidopsis thaliana* seedlings growing in soil. (a–d) The effect of multifactorial stress conditions (heat, salt, excess light, acidity, heavy metal, and oxidative stresses) applied in different combinations (up to a combination of all six factors) was determined on the survival (a) seedling diameter (b), chlorophyll content (c) and whole‐plant ROS levels (d) of wild‐type, *rbohD* and *apx1* seedlings growing in peat soil. Box plots show the median (horizontal line), the lower and upper bounds of each box plot denote the first and third quartiles (the 25^th^ and 75^th^ percentiles, respectively), and whiskers above and below the box plot indicate 1.5 times the interquartile range. Statistical analysis was performed using two‐way ANOVA followed using a Tukey post hoc test (different letters denote statistical significance at *P* < 0.05; Table S50). Abbreviations: Apx1, ascorbate peroxidase 1; Chl, chlorophyll; RbohD, respiratory burst oxidase homologue D; TRE, total radiant efficiency.

Although the study of seedlings growing on plates enabled us to precisely analyse plant survival and root growth in response to different multifactorial stress combinations (Figs 1, 2, 3, 4, 5, 6, S1–S6), the responses of plants grown on plates may not always reflect those of plants grown in soil (Mittler & Blumwald, 2010). We therefore subjected Arabidopsis wild‐type, *apx1* and *rbohD* seedlings grown in peat soil to the same multifactorial stress combinations as those grown on plates. As shown in Figs 7 and  S7–S9, the response of peat soil‐grown wild‐type and *apx1* seedlings to the multifactorial stress combination was similar to that of plants grown on plates, with *apx1* seedlings demonstrating a significant decrease in growth and survival, coupled with an increase in overall ROS levels, compared with wild‐type, in response to all six stresses combined (Figs 1, 7, S1–S4, S7–S9). By contrast, although *rbohD* displayed a similar decline to that of wild‐type and *apx1* in growth and survival, and a similar increase in overall ROS levels, in response to the increasing number and complexity of multifactorial stress combinations, compared with its survival on plates, the impact of the multifactorial stress combination on *rbohD* was not as severe in peat soil (Figs 1, 7, S1–S4, S7–S9). Compared with seedlings grown on plates (Fig. 1a), the overall survival of seedlings grown in peat soil (Fig. 7a) was higher in response to the different stresses and their combinations. Although it is hard to draw conclusions from such a comparison, it is possible that the presence of the plant microbiome (e.g. De Vries *et al*., 2020; Liu *et al*., 2020), and/or the buffering effects of the peat soil on the different stressors (i.e. altering the pH, or binding of Cd or PQ), enhanced the ability of seedlings to withstand different abiotic stresses and their combinations. Taken together, the results shown in Figs 1 and 7, and Figs S1–S4 and S7–S9, demonstrate that multifactorial stress combinations have a similar overall impact on plants grown in peat soil (Figs 7, S7–S9) or on plates (Figs 1, S1–S4), and that the role of ROS scavenging, mediated by APX1, is important for plant survival under both conditions.

## Discussion

While each of the different stresses, applied individually, had a minimal effect on plant growth and survival, the accumulated impact of multifactorial stress combination on plants, growing both in peat soil and on plates, was detrimental (Figs 1, 7, S1–S4,S7–S9). This finding is important as it demonstrates that different stresses could interact to negatively impact plant health and performance, even if the effect of each stress applied individually is negligible. A multifactorial stress combination could therefore impact an ecosystem or an agricultural area in ways that we may not be able to currently predict. For example, we may not observe a clear decline in an ecosystem or a field due to a low level of one stress factor, but once additional factors are introduced, even at low levels, they could negatively interact with each other and push the system towards a rapid collapse. Together with the pioneering study of Rillig *et al*., (2019), our results therefore suggest that with the increasing number and complexity of simultaneously occurring environmental stress factors on our planet, plant life (Figs 1, 7, S1–S4, S7–S9) as well as soils (Rillig *et al*., 2019), are likely to deteriorate further. The similar trends observed in our study (Figs 1, 7, S1–S4, S7–S9) and that of Rillig *et al*. (2019), should serve as a dire warning to our society. Further polluting our environment could result in even higher complexities of multifactorial stress combinations that in turn would drive a critical decline in plant growth, soil conditions and overall agricultural productivity.

The combined phenotypic and representative gene expression analyses presented by our study (Figs 1, 2, 3, 4, 5, 6, 7, S1–S9) further highlight the uniqueness of plant responses to stress combination (Rizhsky *et al*., 2004; Mittler, 2006; Mittler & Blumwald, 2010; Prasch & Sonnewald, 2013; Suzuki *et al*., 2014; Choudhury *et al*., 2017; Shaar‐Moshe *et al*., 2017, 2019; Zhang & Sonnewald, 2017; Balfagón *et al*., 2019; Zandalinas *et al*., 2020a), even at the multifactorial level. The identification of six‐stress combination‐specific gene expression patterns included within the representative set of multifactorial stress conditions studied (Tables S39–S41) suggests for example that even in response to a combination of six different stresses, certain aspects of the plant response are likely to be unique and cannot be predicted from the response of plants to different stress combinations applied as four‐ or five‐factor stresses. It is surprising that even under such a high level of stress complexity, distinct gene expression signatures can be identified, suggesting that each different combination of stresses is unique in its effects on plant metabolism, physiology and survival and requires a unique gene expression response for plant acclimation.

Our study further reveals that maintaining two critical biological processes, namely, iron and ROS homeostasis, is essential for plant acclimation to multifactorial stress combinations (Figs 1, 2, 3, 7, S1–S9). Balancing iron and ROS levels is thought to be essential for the survival of different microorganisms growing under extreme environmental conditions (Slade & Radman, 2011; Yuan *et al*., 2012; Schieber & Chandel, 2014; Halliwell & Gutteridge, 2015; Mittler, 2017; Shuryak, 2019), providing further support to our findings with plants. The observation that plants overexpressing AtNEET, a protein essential for the management of iron and ROS in plant and animal cells (Nechushtai *et al*., 2012; Darash‐Yahana *et al*., 2016; Mittler *et al*., 2019; Zandalinas *et al*., 2020b) could maintain root growth under conditions of multifactorial stress combinations (Figs 3d, S6), and that an RNAi line for AtNEET is highly sensitive to multifactorial stress combinations (Fig. 6), lends further support to this hypothesis.

In addition to iron and ROS metabolism, other cellular processes such as autophagy, hormone signalling (in particular ABA), heat stress responses (in particular MBF1c‐regulated), DNA repair and osmoregulation are likely to be important for plant acclimation to multifactorial stress combinations (Fig. 6). Interestingly, not all stresses and their combinations resulted in the activation/suppression of these pathways to a similar extent. Of particular interest are the combinations of PQ + HL + HS, Salt + PQ + HL and Salt + PQ + HL + HS, that appear to involve a lower proportion of transcripts involved in many of the pathways activated by the other stresses (Fig. 5). Interestingly, the percentage of unique genes activated by these specific combinations (PQ + HL + HS, Salt + PQ + HL and Salt + PQ + HL + HS) was also higher compared with those found to be triggered by all other stresses (Fig. 4b), suggesting that the response of Arabidopsis to these particular combinations is unique and may involve pathways or metabolites with a defence/acclimation role, not identified/studied yet. This possibility is further supported by the hierarchical clustering analysis shown in Fig. 2, which reveals distinct clustering of genes expressed in response to PQ + HL + HS, Salt + PQ + HL, and Salt + PQ + HL + HS, among all gene expression patterns common to all three, four, five and six‐stress combinations. Although further studies are needed to address this possibility, our findings highlight the unique impact of multifactorial stress combination on plants and its effect on the regulation of multiple stress‐response pathways in Arabidopsis.

In addition to impacting plant growth and survival (Figs 1, 7), multifactorial stress combinations are also likely to impact plant reproduction and different biotic interactions (not addressed in this study). In this respect it should be noted that reproductive processes and yield of important grain crops such as corn (*Zea mays*), soybean (*Glycine max*) and wheat (*Triticum aestivum*) are negatively impacted by stress combinations such as drought and heat stress (e.g. Mittler, 2006; Li *et al*., 2015; Lawas *et al*., 2018; Qaseem *et al*., 2019; Cohen *et al*., 2021). In addition, plant–pathogen and/or insect interactions are also negatively impacted by different stresses and their combinations (e.g. Prasch & Sonnewald, 2013; Desaint *et al*., 2020; Hamann *et al*., 2020; Cohen & Leach, 2020; Savary & Willocquet, 2020). If simple stress combinations involving two or at most three factors can have such dramatic effects on plant reproduction and/or pathogen/insect interactions, it stands to reason that more complex stress interactions, such as those comprising a multifactorial stress combination, would have an even more dramatic effect on these processes. Considering the increased rate of changes in global environmental conditions, further studies that address the impacts of multifactorial stress combination on reproductive processes and yield could prove critical for our global food, feed and fibre security.

Taken together, our findings demonstrated that, with the increasing number and complexity of multifactorial stress combinations, plant growth and survival declines. This decline is evident in the presence (Figs 7, S7–S9) or absence (Figs 1, S1–S4) of soil, that is potentially also impacted by multifactorial stress conditions (Rillig *et al*., 2019), and is dependent on the ability of plants to scavenge ROS (Figs 1, 7, S1–S4, S6–S9), manage iron levels (Figs 3, 5), mediate ABA signalling (Figs 3a, 5), and mount a heat stress response utilizing MBF1c (Fig. 6). Although the study of multifactorial stress combination in plants is in its infancy, it could potentially lead to new and exciting discoveries, as well as reveal new strategies to mitigate the impact of multifactorial stress conditions on our eco‐ and agricultural systems that are facing a growing challenge due to global climatic changes and human interventions.

## Author contributions

SIZ and SS performed experiments and analysed the data. RM, FBF and SIZ designed experiments and analysed the data. RKA coordinated bioinformatics analysis. RM, SIZ, FBF, RKA and RN wrote the manuscript. All authors read and approved the manuscript.

## Supporting information


**Fig. S1** Survival of Arabidopsis wild‐type, *rbohD* and *apx1* seedlings subjected to multifactorial stress combinations of heat, salt, light, oxidative stresses, acidity and cadmium.
**Fig. S2** Total and delta (Δ) root growth of Arabidopsis wild‐type, *rbohD* and *apx1* seedlings subjected multifactorial stress combinations of heat, salt, light, oxidative stresses, acidity and cadmium.
**Fig. S3** Chlorophyll content of Arabidopsis wild‐type, *rbohD* and *apx1* seedlings subjected to multifactorial stress combinations of heat, salt, light, oxidative stresses, acidity and cadmium.
**Fig. S4** Whole‐plant ROS accumulation of Arabidopsis wild‐type, *rbohD* and *apx1* seedlings subjected to multifactorial stress combinations of heat, salt, light, oxidative stresses, acidity and cadmium.
**Fig. S5** Gene expression analysis of multifactorial stress responses.
**Fig. S6** Total and delta (Δ) root growth, and whole‐plant ROS accumulation of Arabidopsis wild‐type and AtNEET seedlings subjected to multifactorial stress combinations of heat, salt, light and oxidative stresses applied in all possible combinations.
**Fig. S7** Survival and seedling diameter of Arabidopsis wild‐type, *rbohD* and *apx1* seedlings growing in soil subjected to multifactorial stress combinations of heat, salt, light, oxidative stresses, acidity and cadmium.
**Fig.** **S8** Chlorophyll content of Arabidopsis wild‐type, *rbohD* and *apx1* seedlings growing in soil subjected to multifactorial stress combinations of heat, salt, light, oxidative stresses, acidity and cadmium.
**Fig.** **S9** Whole‐plant ROS accumulation of Arabidopsis wild‐type, *rbohD* and *apx1* seedlings growing in soil subjected to multifactorial stress combinations of heat, salt, light, oxidative stresses, acidity and cadmium.

**Table S1** Genes significantly upregulated compared with control (*P* < 0.05) in Col seedlings subjected to salt stress.
**Table S2** Genes significantly upregulated compared with control (*P* < 0.05) in Col seedlings subjected to paraquat.
**Table S3** Genes significantly upregulated compared with control (*P* < 0.05) in Col seedlings subjected to high light stress.
**Table S4** Genes significantly upregulated compared with control (*P* < 0.05) in Col seedlings subjected to heat stress.
**Table S5** Genes significantly upregulated compared with control (*P* < 0.05) in Col seedlings subjected to salt + high light stress combination.
**Table S6** Genes significantly upregulated compared with control (*P* < 0.05) in Col seedlings subjected to paraquat + high light stress combination.
**Table S7** Genes significantly upregulated compared with control (*P* < 0.05) in Col seedlings subjected to salt + heat stress combination.
**Table S8** Genes significantly upregulated compared with control (*P* < 0.05) in Col seedlings subjected to paraquat + heat stress combination.
**Table S9** Genes significantly upregulated compared with control (*P* < 0.05) in Col seedlings subjected to salt + paraquat stress combination.
**Table S10** Genes significantly upregulated compared with control (*P* < 0.05) in Col seedlings subjected to high light + heat stress combination.
**Table S11** Genes significantly upregulated compared with control (*P* < 0.05) in Col seedlings subjected to salt + paraquat + high light stress combination.
**Table S12** Genes significantly upregulated compared with control (*P* < 0.05) in Col seedlings subjected to salt + paraquat + heat stress combination.
**Table S13** Genes significantly upregulated compared with control (*P* < 0.05) in Col seedlings subjected to salt + high light + heat stress combination.
**Table S14** Genes significantly upregulated compared with control (*P* < 0.05) in Col seedlings subjected to paraquat + high light + heat stress combination.
**Table S15** Genes significantly upregulated compared with control (*P* < 0.05) in Col seedlings subjected to paraquat + salt + high light + heat stress combination.
**Table S16** Genes significantly upregulated compared with control (*P* < 0.05) in Col seedlings subjected to paraquat + salt + high light + heat stress + acid combination.
**Table S17** Genes significantly upregulated compared with control (*P* < 0.05) in Col seedlings subjected to paraquat + salt + high light + heat stress + cadmium combination.
**Table S18** Genes significantly upregulated compared with control (*P* < 0.05) in Col seedlings subjected to paraquat + salt + high light + heat stress + acid + cadmium combination.
**Table S19** Genes significantly downregulated compared with control (*P* < 0.05) in Col seedlings subjected to salt stress.
**Table S20** Genes significantly downregulated compared with control (*P* < 0.05) in Col seedlings subjected to paraquat.
**Table S21** Genes significantly downregulated compared with control (*P* < 0.05) in Col seedlings subjected to high light stress.
**Table S22** Genes significantly downregulated compared with control (*P* < 0.05) in Col seedlings subjected to heat stress.
**Table S23** Genes significantly downregulated compared with control (*P* < 0.05) in Col seedlings subjected to salt + high light stress combination.
**Table S24** Genes significantly downregulated compared with control (*P* < 0.05) in Col seedlings subjected to paraquat + high light stress combination.
**Table S25** Genes significantly downregulated compared with control (*P* < 0.05) in Col seedlings subjected to salt + heat stress combination.
**Table S26** Genes significantly downregulated compared with control (*P* < 0.05) in Col seedlings subjected to paraquat + heat stress combination.
**Table S27** Genes significantly downregulated compared with control (*P* < 0.05) in Col seedlings subjected to salt + paraquat stress combination.
**Table S28** Genes significantly downregulated compared with control (*P* < 0.05) in Col seedlings subjected to high light + heat stress combination.
**Table S29** Genes significantly downregulated compared with control (*P* < 0.05) in Col seedlings subjected to salt + paraquat + high light stress combination.
**Table S30** Genes significantly downregulated compared with control (*P* < 0.05) in Col seedlings subjected to salt + paraquat + heat stress combination.
**Table S31** Genes significantly downregulated compared with control (*P* < 0.05) in Col seedlings subjected to salt + high light + heat stress combination.
**Table S32** Genes significantly downregulated compared with control (*P* < 0.05) in Col seedlings subjected to paraquat + high light + heat stress combination.
**Table S33** Genes significantly downregulated compared with control (*P* < 0.05) in Col seedlings subjected to paraquat + salt + high light + heat stress combination.
**Table S34** Genes significantly downregulated compared with control (*P* < 0.05) in Col seedlings subjected to paraquat + salt + high light + heat stress + acid combination.
**Table S35** Genes significantly downregulated compared with control (*P* < 0.05) in Col seedlings subjected to paraquat + salt + high light + heat stress + cadmium combination.
**Table S36** Genes significantly downregulated compared with control (*P* < 0.05) in Col seedlings subjected to paraquat + salt + high light + heat stress + acid + cadmium combination.
**Table S37** List of genes common between genes upregulated in all four‐stress possible three‐stress combinations and genes upregulated in response to all four, five and six stresses combined (Fig. 2).
**Table S38** List of genes common between genes downregulated in all four possible three‐stress combinations and genes downregulated in response to all four, five and six stresses combined (Fig. S5).
**Table S39** List of significantly up and downregulated genes unique to each stress condition.
**Table S40** List of significantly upregulated genes unique to the state of six‐stress combination.
**Table S41** List of significantly downregulated genes unique to the state of six‐stress combination.
**Table S42** List of genes involved in chlorophyll metabolism.
**Table S43** List of genes involved in osmoregulation metabolism.
**Table S44** List of genes involved in autophagy.
**Table S45** List of genes involved in DNA repair.
**Table S46** List of genes involved in proteolysis.
**Table S47** List of genes involved in senescence.
**Table S48** List of heat shock factor (HSF) genes.
**Table S49** List of genes involved in unfolded protein response (UPR).
**Table S50** *P*‐values for Figs 1–7.
Please note: Wiley Blackwell are not responsible for the content or functionality of any Supporting Information supplied by the authors. Any queries (other than missing material) should be directed to the *New Phytologist* Central Office.
