# Supplementary material for: The impact of multifactorial stress combination on plant growth and survival
Source: New Phytol. 2021 Feb 18;230(3):1034–48. doi: 10.1111/nph.17232 (PMC8048544; doi:10.1111/nph.17232)
Supplement: Supplementary file 1 — Fig. S1 Survival of Arabidopsis wild‐type, rbohD and apx1 seedlings subjected to multifactorial stress combinations of heat, salt, light, oxidative stresses, acidity and cadmium. Fig. S2 Total and delta (Δ) root growth of Arabidopsis wild‐type, rbohD and apx1 seedlings subjected multifactorial stress combinations of heat, salt, light, oxidative stresses, acidity and cadmium. Fig. S3 Chlorophyll content of Arabidopsis wild‐type, rbohD and apx1 seedlings subjected to multifactorial stress combinations of heat, salt, light, oxidative stresses, acidity and cadmium. Fig. S4 Whole‐plant ROS accumulation of Arabidopsis wild‐type, rbohD and apx1 seedlings subjected to multifactorial stress combinations of heat, salt, light, oxidative stresses, acidity and cadmium. Fig. S5 Gene expression analysis of multifactorial stress responses. Fig. S6 Total and delta (Δ) root growth, and whole‐plant ROS accumulation of Arabidopsis wild‐type and AtNEET seedlings subjected to multifactorial stress combinations of heat, salt, light and oxidative stresses applied in all possible combinations. Fig. S7 Survival and seedling diameter of Arabidopsis wild‐type, rbohD and apx1 seedlings growing in soil subjected to multifactorial stress combinations of heat, salt, light, oxidative stresses, acidity and cadmium. Fig. S8 Chlorophyll content of Arabidopsis wild‐type, rbohD and apx1 seedlings growing in soil subjected to multifactorial stress combinations of heat, salt, light, oxidative stresses, acidity and cadmium. Fig. S9 Whole‐plant ROS accumulation of Arabidopsis wild‐type, rbohD and apx1 seedlings growing in soil subjected to multifactorial stress combinations of heat, salt, light, oxidative stresses, acidity and cadmium. [file NPH-230-1034-s002.pdf]

New Phytologist

***New Phytologist* Supporting Information**

Article title: The impact of multifactorial stress combination on plant growth and survival
Authors: Sara I. Zandalinas, Soham Sengupta, Felix B. Fritschi, Rajeev K. Azad, Rachel Nechushtai and Ron Mittler
Article acceptance date: 17 January 2021

The following Supporting Information is available for this article:

Fig. S1. Survival of Arabidopsis wildtype, *rbohD* and *apx1* seedlings subjected to multifactorial stress combinations of heat, salt, light, oxidative stresses, acidity and cadmium. Results are presented as the mean ± SD. Statistical analysis was performed by two-way ANOVA followed by a Tukey post hoc test (asterisks denote statistical significance at $p < 0.05$ with respect to controls). Abbreviations: Apx1, ascorbate peroxidase 1; RbohD, respiratory burst oxidase homolog D; CT, control; PQ, paraquat; HL, high light; HS, heat stress.

Fig. S2. Total and delta (Δ) root growth of Arabidopsis wildtype, *rbohD* and *apx1* seedlings subjected multifactorial stress combinations of heat, salt, light, oxidative stresses, acidity and cadmium. Results are presented as the mean ± SD. Statistical analysis was performed by two-way ANOVA followed by a Tukey post hoc test (asterisks denote statistical significance at $p < 0.05$ with respect to controls). Abbreviations: Apx1, ascorbate peroxidase 1; RbohD, respiratory burst oxidase homolog D; CT, control; PQ, paraquat; HL, high light; HS, heat stress.

Fig. S3. Chlorophyll content of Arabidopsis wildtype, *rbohD* and *apx1* seedlings subjected to multifactorial stress combinations of heat, salt, light, oxidative stresses, acidity and cadmium. Results are presented as the mean ± SD. Statistical analysis was performed by two-way ANOVA followed by a Tukey post hoc test (asterisks denote statistical significance at $p < 0.05$ with respect to controls for total chlorophyll). Abbreviations: Apx1, ascorbate peroxidase 1; RbohD, respiratory burst oxidase homolog D; Chl, chlorophyll; CT, control; PQ, paraquat; HL, high light; HS, heat stress.

Fig. S4. Whole-plant ROS accumulation of Arabidopsis wildtype, *rbohD* and *apx1* seedlings subjected to multifactorial stress combinations of heat, salt, light, oxidative stresses, acidity and cadmium. Results are presented as the mean ± SD. Statistical analysis was performed by two-way ANOVA followed by a Tukey post hoc test (asterisks denote statistical significance at $p < 0.05$ with respect to controls). Abbreviations: Apx1, ascorbate peroxidase 1; RbohD, respiratory burst oxidase homolog D; TRE, Total Radiant Efficiency; CT, control; PQ, paraquat; HL, high light; HS, heat stress.

Fig. S5. Gene expression analysis of multifactorial stress responses. Gene expression analysis of the response of Arabidopsis seedlings to different multifactorial stress combinations of heat, salt, excess light, oxidative stress (induced by the herbicide paraquat), acidity and heavy metal (cadmium) is shown (see also Fig. 2). (a) Venn diagrams depicting the overlap between genes upregulated (left) or downregulated (right) in their expression in response to each of the different single stresses [Salt, paraquat (PQ), high light (HL), or heat stress (HS)]. **(b)** Venn diagrams depicting the overlap between genes upregulated (left) or downregulated (right) in their expression in response to each of the different 2 stress combinations. **(c)** Venn diagrams depicting the overlap between genes downregulated in their expression in response to several different 3 factor stress combinations (left), or 4-, 5- and 6- stress factor combinations (right) are shown on top. A Venn diagram showing the overlap between genes downregulated in their expression in response to several different 3 factor stress combinations and genes downregulated in their expression in response to 4-, 5- and 6- stress factor combinations (127 genes) is shown underneath, together with bar and pie charts of biological process and molecular function (GO) annotations for these genes, and a heat map showing the expression level and clustering of these genes under all treatment combinations tested. **(d)** Venn diagrams depicting the overlap between genes upregulated (left) or downregulated (right) in their expression in response to 3-, 4-, 5- and 6- stress factor combinations (136 and 127 genes, respectively), and genes upregulated (left) or downregulated (right) in their expression in response to each of the different single stresses [Salt, paraquat (PQ), high light (HL), or heat stress (HS)]. Statistical significance of Venn diagrams overlap was determined by hypergeometric testing analysis using

the R package phyper. Abbreviations: A, acidity; Cd, cadmium; HL, high light; HS, heat stress; PQ, paraquat.

Fig. S6. Total and delta (Δ) root growth, and whole-plant ROS accumulation of Arabidopsis wildtype and AtNEET seedlings subjected to multifactorial stress combinations of heat, salt, light and oxidative stresses applied in all possible combinations. Results are presented as the mean ± SD. Statistical analysis was performed by two-way ANOVA followed by a Tukey post hoc test (asterisks denote statistical significance at $p < 0.05$ with respect to controls). Abbreviations: CT, control; PQ, paraquat; HL, high light; HS, heat stress; TRE, Total Radiant Efficiency.

Fig. S7. Survival and seedling diameter of Arabidopsis wildtype, *rbohD* and *apx1* seedlings growing in soil subjected to multifactorial stress combinations of heat, salt, light, oxidative stresses, acidity and cadmium. Results are presented as the mean ± SD. Statistical analysis was performed by two-way ANOVA followed by a Tukey post hoc test (asterisks denote statistical significance at $p < 0.05$ with respect to controls). Abbreviations: Apx1, ascorbate peroxidase 1; RbohD, respiratory burst oxidase homolog D; CT, control; HL, high light; HS, heat stress; PQ, paraquat.

Fig. S8. Chlorophyll content of Arabidopsis wildtype, *rbohD* and *apx1* seedlings growing in soil subjected to multifactorial stress combinations of heat, salt, light, oxidative stresses, acidity and cadmium. Results are presented as the mean ± SD. Statistical analysis was performed by two-way ANOVA followed by a Tukey post hoc test (asterisks denote statistical significance at $p < 0.05$ with respect to controls). Abbreviations: Apx1, ascorbate peroxidase 1; RbohD, respiratory burst oxidase homolog D; Chl, chlorophyll; CT, control; HL, high light; HS, heat stress; PQ, paraquat.

Fig. S9. Whole-plant ROS accumulation of Arabidopsis wildtype, *rbohD* and *apx1* seedlings growing in soil subjected to multifactorial stress combinations of heat, salt, light, oxidative stresses, acidity and cadmium. Results are presented as the mean ± SD. Statistical analysis was performed by two-way ANOVA followed by a Tukey post hoc test (asterisks denote statistical significance at $p < 0.05$ with respect to controls). Abbreviations: Apx1, ascorbate peroxidase 1;

RbohD, respiratory burst oxidase homolog D; TRE, Total Radiant Efficiency; CT, control; PQ, paraquat; HL, high light; HS, heat stress.

Table S1. Genes significantly upregulated compared to control ($p < 0.05$) in Col seedlings subjected to salt stress.

Table S2. Genes significantly upregulated compared to control ($p < 0.05$) in Col seedlings subjected to paraquat.

Table S3. Genes significantly upregulated compared to control ($p < 0.05$) in Col seedlings subjected to high light stress.

Table S4. Genes significantly upregulated compared to control ($p < 0.05$) in Col seedlings subjected to heat stress.

Table S5. Genes significantly upregulated compared to control ($p < 0.05$) in Col seedlings subjected to salt + high light stress combination.

Table S6. Genes significantly upregulated compared to control ($p < 0.05$) in Col seedlings subjected to paraquat + high light stress combination.

Table S7. Genes significantly upregulated compared to control ($p < 0.05$) in Col seedlings subjected to salt + heat stress combination.

Table S8. Genes significantly upregulated compared to control ($p < 0.05$) in Col seedlings subjected to paraquat + heat stress combination.

Table S9. Genes significantly upregulated compared to control ($p < 0.05$) in Col seedlings subjected to salt + paraquat stress combination.

Table S10. Genes significantly upregulated compared to control ($p < 0.05$) in Col seedlings subjected to high light + heat stress combination.

Table S11. Genes significantly upregulated compared to control ($p < 0.05$) in Col seedlings subjected to salt + paraquat + high light stress combination.

Table S12. Genes significantly upregulated compared to control ($p < 0.05$) in Col seedlings subjected to salt + paraquat + heat stress combination.

Table S13. Genes significantly upregulated compared to control ($p < 0.05$) in Col seedlings subjected to salt + high light + heat stress combination.

Table S14. Genes significantly upregulated compared to control ($p < 0.05$) in Col seedlings subjected to paraquat + high light + heat stress combination.

Table S15. Genes significantly upregulated compared to control ($p < 0.05$) in Col seedlings subjected to paraquat + salt + high light + heat stress combination.

Table S16. Genes significantly upregulated compared to control ($p < 0.05$) in Col seedlings subjected to paraquat + salt + high light + heat stress + acid combination.

Table S17. Genes significantly upregulated compared to control ($p < 0.05$) in Col seedlings subjected to paraquat + salt + high light + heat stress + cadmium combination.

Table S18. Genes significantly upregulated compared to control ($p < 0.05$) in Col seedlings subjected to paraquat + salt + high light + heat stress + acid + cadmium combination.

Table S19. Genes significantly downregulated compared to control ($p < 0.05$) in Col seedlings subjected to salt stress.

Table S20. Genes significantly downregulated compared to control ($p < 0.05$) in Col seedlings subjected to paraquat.

Table S21. Genes significantly downregulated compared to control ($p < 0.05$) in Col seedlings subjected to high light stress.

Table S22. Genes significantly downregulated compared to control ($p < 0.05$) in Col seedlings subjected to heat stress.

Table S23. Genes significantly downregulated compared to control ($p < 0.05$) in Col seedlings subjected to salt + high light stress combination.

Table S24. Genes significantly downregulated compared to control ($p < 0.05$) in Col seedlings subjected to paraquat + high light stress combination.

Table S25. Genes significantly downregulated compared to control ($p < 0.05$) in Col seedlings subjected to salt + heat stress combination.

Table S26. Genes significantly downregulated compared to control ($p < 0.05$) in Col seedlings subjected to paraquat + heat stress combination.

Table S27. Genes significantly downregulated compared to control ($p < 0.05$) in Col seedlings subjected to salt + paraquat stress combination.

Table S28. Genes significantly downregulated compared to control ($p < 0.05$) in Col seedlings subjected to high light + heat stress combination.

Table S29. Genes significantly downregulated compared to control ($p < 0.05$) in Col seedlings subjected to salt + paraquat + high light stress combination.

Table S30. Genes significantly downregulated compared to control ($p < 0.05$) in Col seedlings subjected to salt + paraquat + heat stress combination.

Table S31. Genes significantly downregulated compared to control ($p < 0.05$) in Col seedlings subjected to salt + high light + heat stress combination.

Table S32. Genes significantly downregulated compared to control ($p < 0.05$) in Col seedlings subjected to paraquat + high light + heat stress combination.

Table S33. Genes significantly downregulated compared to control ($p < 0.05$) in Col seedlings subjected to paraquat + salt + high light + heat stress combination.

Table S34. Genes significantly downregulated compared to control ($p < 0.05$) in Col seedlings subjected to paraquat + salt + high light + heat stress + acid combination.

Table S35. Genes significantly downregulated compared to control ($p < 0.05$) in Col seedlings subjected to paraquat + salt + high light + heat stress + cadmium combination.

Table S36. Genes significantly downregulated compared to control ($p < 0.05$) in Col seedlings subjected to paraquat + salt + high light + heat stress + acid + cadmium combination.

Table S37. List of genes common between genes upregulated in all four possible 3 stress combinations and genes upregulated in response to all 4, 5 and 6 stresses combined (Fig. 2).

Table S38. List of genes common between genes downregulated in all four possible 3 stress combinations and genes downregulated in response to all 4, 5 and 6 stresses combined (Fig. S5).

Table S39. List of significantly up and downregulated genes unique to each stress condition.

Table S40. List of significantly upregulated genes unique to the state of six stress combination.

Table S41. List of significantly downregulated genes unique to the state of six stress combination.

Table S42. List of genes involved in chlorophyll metabolism.

Table S43. List of genes involved in osmoregulation metabolism.

Table S44. List of genes involved in autophagy.

Table S45. List of genes involved in DNA repair.

Table S46. List of genes involved in proteolysis.

Table S47. List of genes involved in senescence.

Table S48. List of Heat Shock Factor (HSF) genes.

Table S49. List of genes involved in unfolded protein response (UPR).

Table S50. P-values for Figs. 1-7.

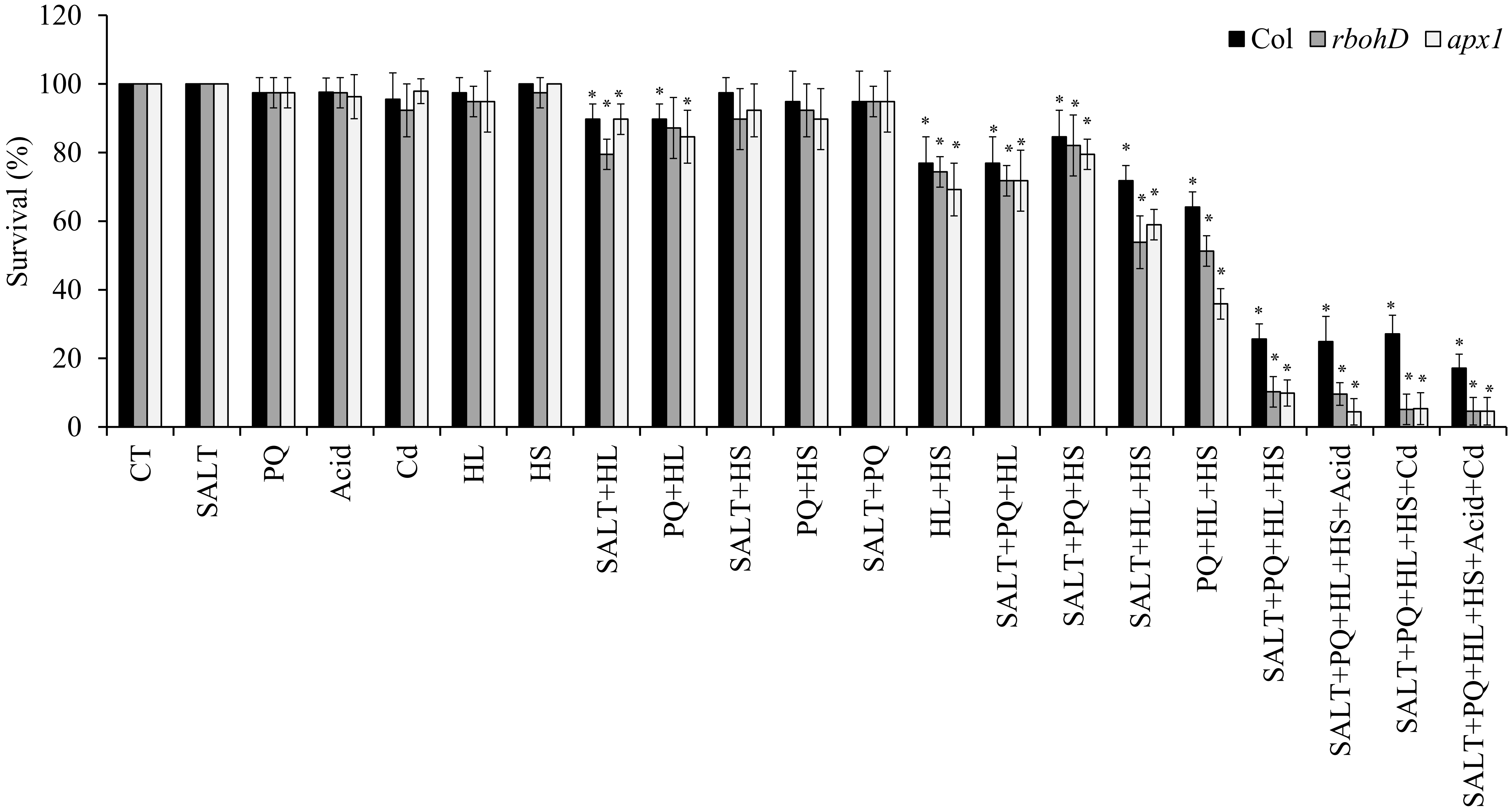

Fig. S1. Survival of Arabidopsis wildtype, *rbohD* and *apx1* seedlings subjected to multifactorial stress combinations of heat, salt, light, oxidative stresses, acidity and cadmium. Results are presented as the mean ± SD. Statistical analysis was performed by two-way ANOVA followed by a Tukey post hoc test (asterisks denote statistical significance at $p < 0.05$ with respect to controls). Abbreviations: Apx1, ascorbate peroxidase 1; RbohD, respiratory burst oxidase homolog D; CT, control; PQ, paraquat; HL, high light; HS, heat stress.

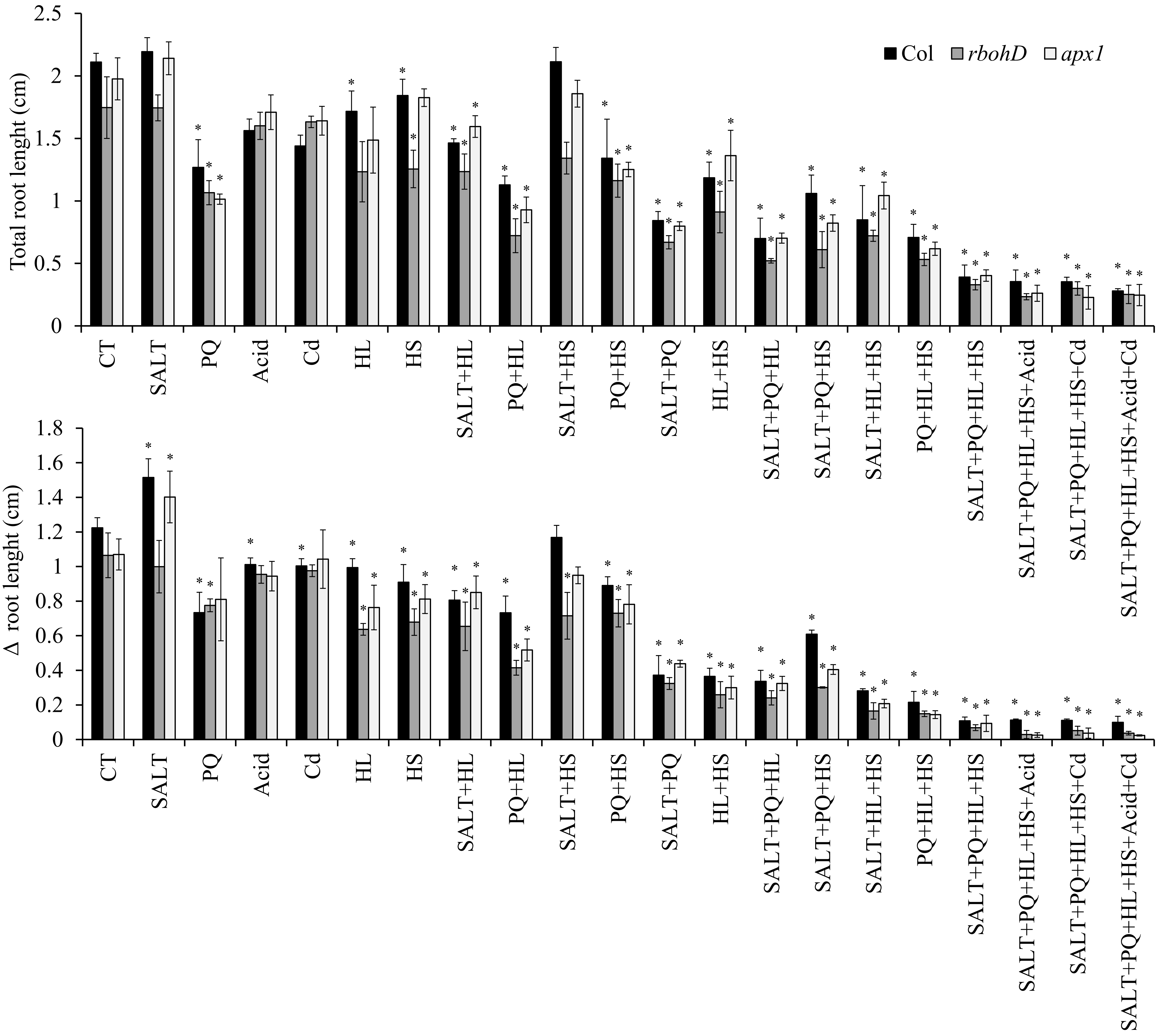

Fig. S2. Total and delta (Δ) root growth of Arabidopsis wildtype, *rbohD* and *apx1* seedlings subjected multifactorial stress combinations of heat, salt, light, oxidative stresses, acidity and cadmium. Results are presented as the mean ± SD. Statistical analysis was performed by two-way ANOVA followed by a Tukey post hoc test (asterisks denote statistical significance at $p < 0.05$ with respect to controls). Abbreviations: Apx1, ascorbate peroxidase 1; RbohD, respiratory burst oxidase homolog D; CT, control; PQ, paraquat; HL, high light; HS, heat stress.

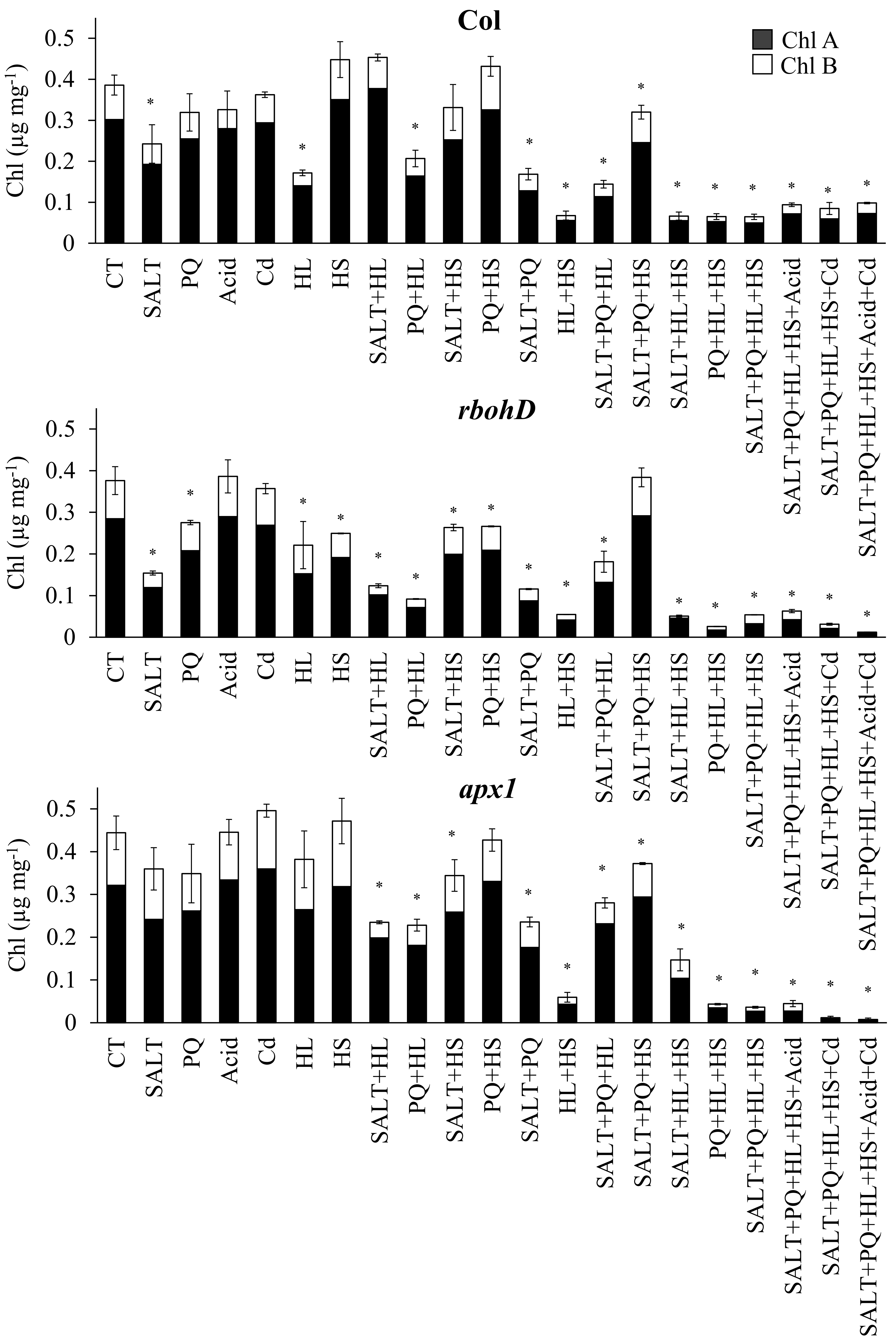

Fig. S3. Chlorophyll content of Arabidopsis wildtype, *rbohD* and *apx1* seedlings subjected to multifactorial stress combinations of heat, salt, light, oxidative stresses, acidity and cadmium. Results are presented as the mean ± SD. Statistical analysis was performed by two-way ANOVA followed by a Tukey post hoc test (asterisks denote statistical significance at $p < 0.05$ with respect to controls for total chlorophyll). Abbreviations: Apx1, ascorbate peroxidase 1; RbohD, respiratory burst oxidase homolog D; Chl, chlorophyll; CT, control; PQ, paraquat; HL, high light; HS, heat stress.

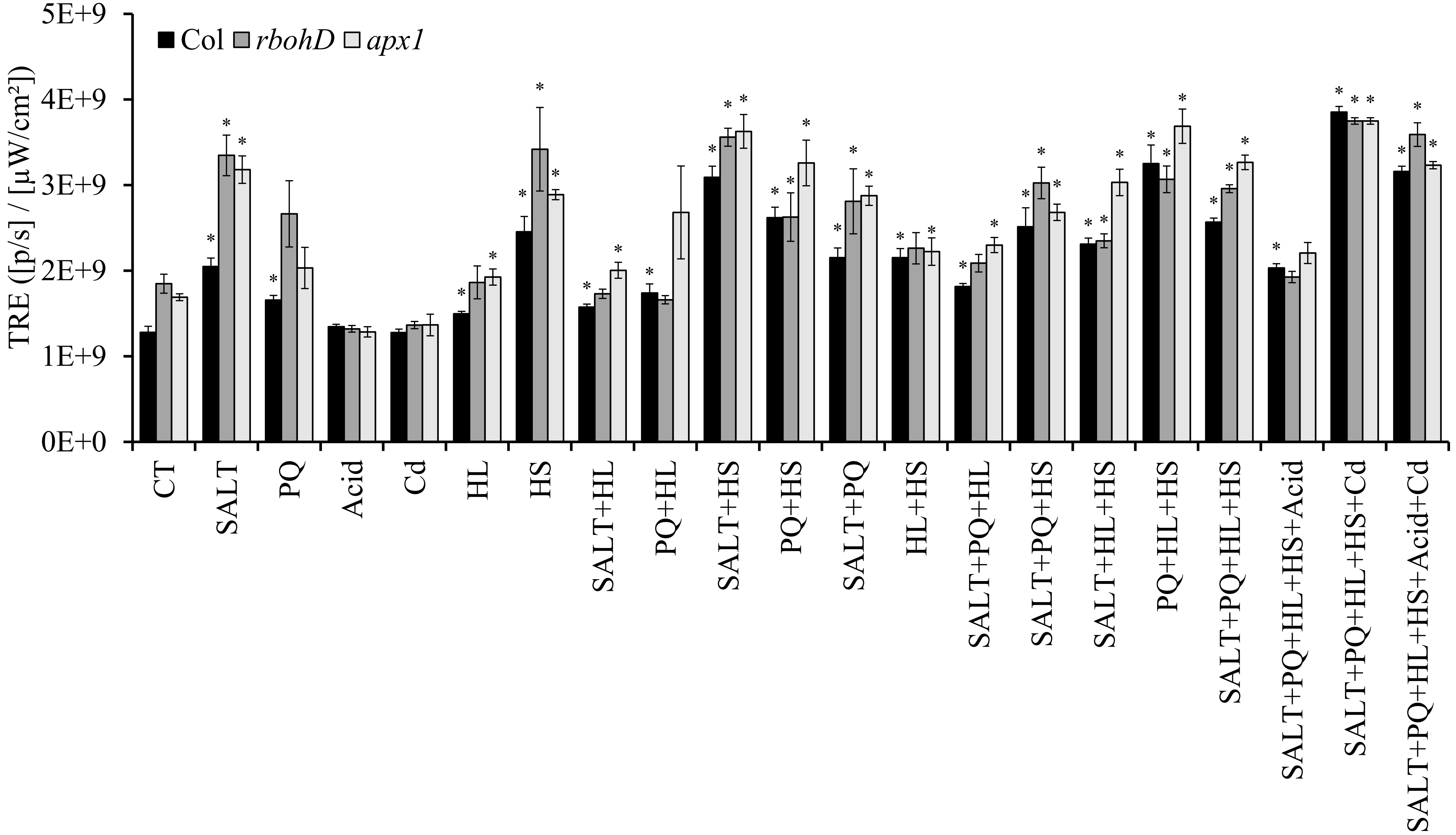

Fig. S4. Whole-plant ROS accumulation of Arabidopsis wildtype, *rbohD* and *apx1* seedlings subjected to multifactorial stress combinations of heat, salt, light, oxidative stresses, acidity and cadmium. Results are presented as the mean ± SD. Statistical analysis was performed by two-way ANOVA followed by a Tukey post hoc test (asterisks denote statistical significance at $p < 0.05$ with respect to controls). Abbreviations: Apx1, ascorbate peroxidase 1; RbohD, respiratory burst oxidase homolog D; TRE, Total Radiant Efficiency; CT, control; PQ, paraquat; HL, high light; HS, heat stress.

(a)

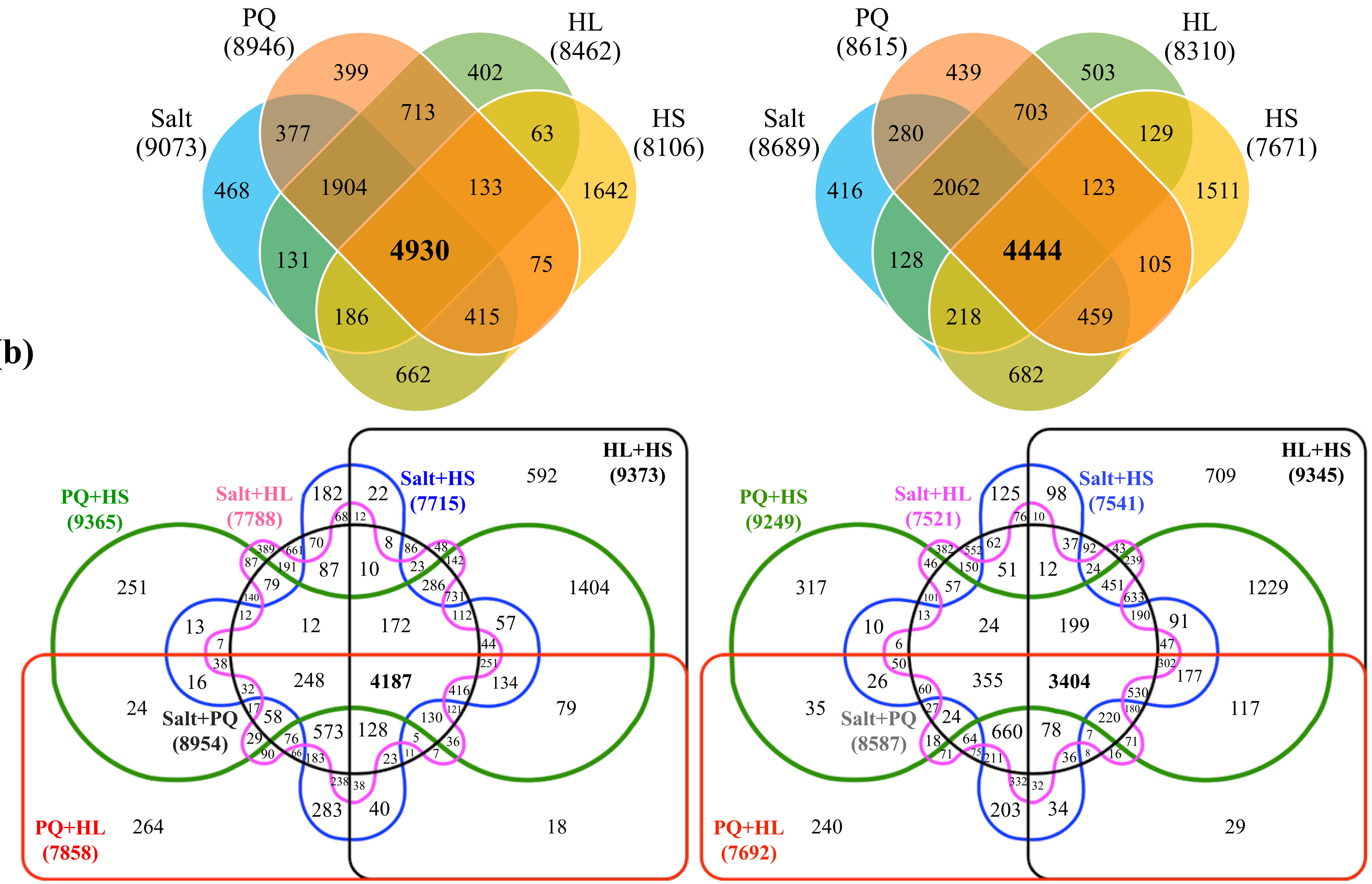

Fig. S5. Gene expression analysis of multifactorial stress responses. Gene expression analysis of the response of Arabidopsis seedlings to different multifactorial stress combinations of heat, salt, excess light, oxidative stress (induced by the herbicide paraquat), acidity and heavy metal (cadmium) is shown (see also Fig. 2). (a) Venn diagrams depicting the overlap between genes upregulated (left) or downregulated (right) in their expression in response to each of the different single stresses [Salt, paraquat (PQ), high light (HL), or heat stress (HS)]. **(b)** Venn diagrams depicting the overlap between genes upregulated (left) or downregulated (right) in their expression in response to each of the different 2 stress combinations. **(c)** Venn diagrams depicting the overlap between genes downregulated in their expression in response to several different 3 factor stress combinations (left), or 4-, 5- and 6- stress factor combinations (right) are shown on top. A Venn diagram showing the overlap between genes downregulated in their expression in response to several different 3 factor stress combinations and genes downregulated in their expression in response to 4-, 5- and 6- stress factor combinations (127 genes) is shown underneath, together with bar and pie charts of biological process and molecular function (GO) annotations for these genes, and a heat map showing the expression level and clustering of these genes under all treatment combinations tested. **(d)** Venn diagrams depicting the overlap between genes upregulated (left) or downregulated (right) in their expression in response to 3-, 4-, 5- and 6- stress factor combinations (136 and 127 genes, respectively), and genes upregulated (left) or downregulated (right) in their expression in response to each of the different single stresses [Salt, paraquat (PQ), high light (HL), or heat stress (HS)]. Statistical significance of Venn diagrams overlap was determined by hypergeometric testing analysis using the R package phyper. Abbreviations: A, acidity; Cd, cadmium; HL, high light; HS, heat stress; PQ, paraquat.

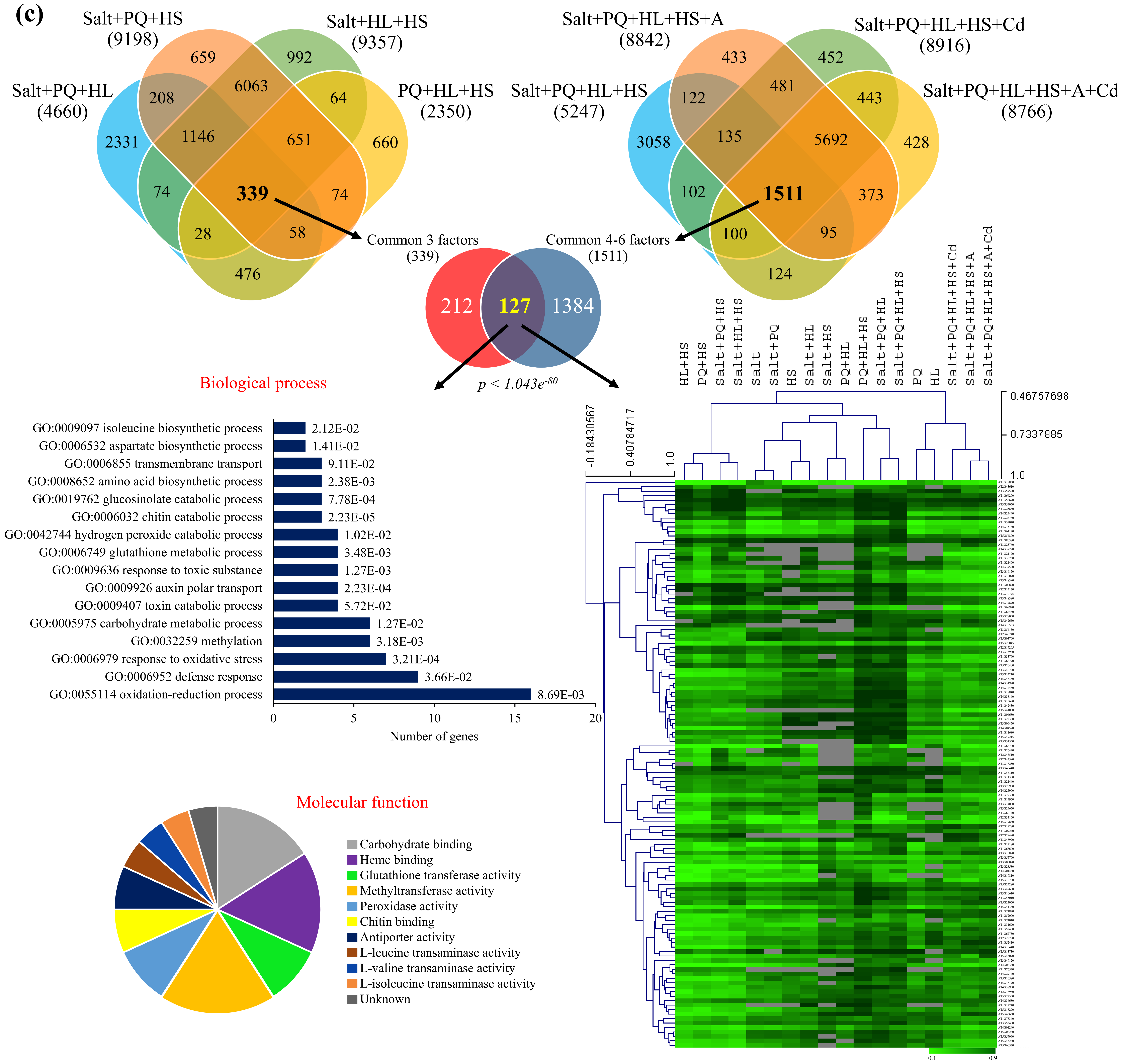

Fig. S5. Gene expression analysis of multifactorial stress responses. Gene expression analysis of the response of Arabidopsis seedlings to different multifactorial stress combinations of heat, salt, excess light, oxidative stress (induced by the herbicide paraquat), acidity and heavy metal (cadmium) is shown (see also Fig. 2). (a) Venn diagrams depicting the overlap between genes upregulated (left) or downregulated (right) in their expression in response to each of the different single stresses [Salt, paraquat (PQ), high light (HL), or heat stress (HS)]. **(b)** Venn diagrams depicting the overlap between genes upregulated (left) or downregulated (right) in their expression in response to each of the different 2 stress combinations. **(c)** Venn diagrams depicting the overlap between genes downregulated in their expression in response to several different 3 factor stress combinations (left), or 4-, 5- and 6- stress factor combinations (right) are shown on top. A Venn diagram showing the overlap between genes downregulated in their expression in response to several different 3 factor stress combinations and genes downregulated in their expression in response to 4-, 5- and 6- stress factor combinations (127 genes) is shown underneath, together with bar and pie charts of biological process and molecular function (GO) annotations for these genes, and a heat map showing the expression level and clustering of these genes under all treatment combinations tested. **(d)** Venn diagrams depicting the overlap between genes upregulated (left) or downregulated (right) in their expression in response to 3-, 4-, 5- and 6- stress factor combinations (136 and 127 genes, respectively), and genes upregulated (left) or downregulated (right) in their expression in response to each of the different single stresses [Salt, paraquat (PQ), high light (HL), or heat stress (HS)]. Statistical significance of Venn diagrams overlap was determined by hypergeometric testing analysis using the R package phyper. Abbreviations: A, acidity; Cd, cadmium; HL, high light; HS, heat stress; PQ, paraquat.

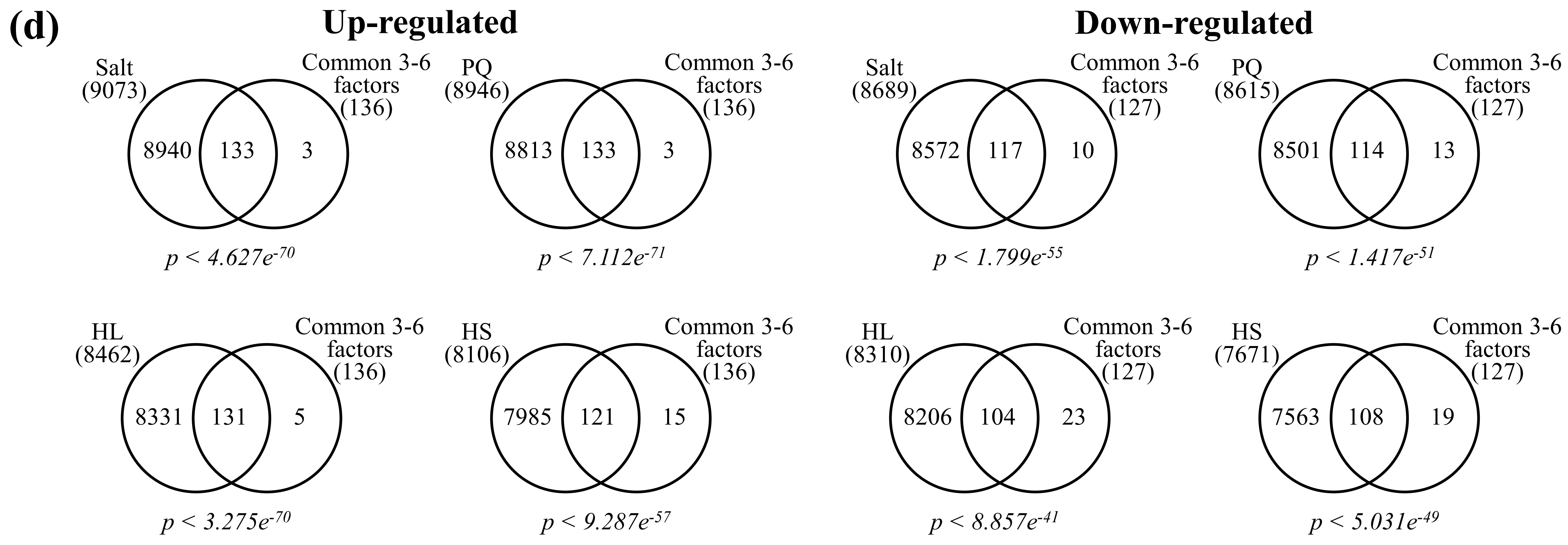

Fig. S5. Gene expression analysis of multifactorial stress responses. Gene expression analysis of the response of Arabidopsis seedlings to different multifactorial stress combinations of heat, salt, excess light, oxidative stress (induced by the herbicide paraquat), acidity and heavy metal (cadmium) is shown (see also Fig. 2). (a) Venn diagrams depicting the overlap between genes upregulated (left) or downregulated (right) in their expression in response to each of the different single stresses [Salt, paraquat (PQ), high light (HL), or heat stress (HS)]. **(b)** Venn diagrams depicting the overlap between genes upregulated (left) or downregulated (right) in their expression in response to each of the different 2 stress combinations. **(c)** Venn diagrams depicting the overlap between genes downregulated in their expression in response to several different 3 factor stress combinations (left), or 4-, 5- and 6- stress factor combinations (right) are shown on top. A Venn diagram showing the overlap between genes downregulated in their expression in response to several different 3 factor stress combinations and genes downregulated in their expression in response to 4-, 5- and 6- stress factor combinations (127 genes) is shown underneath, together with bar and pie charts of biological process and molecular function (GO) annotations for these genes, and a heat map showing the expression level and clustering of these genes under all treatment combinations tested. **(d)** Venn diagrams depicting the overlap between genes upregulated (left) or downregulated (right) in their expression in response to 3-, 4-, 5- and 6- stress factor combinations (136 and 127 genes, respectively), and genes upregulated (left) or downregulated (right) in their expression in response to each of the different single stresses [Salt, paraquat (PQ), high light (HL), or heat stress (HS)]. Statistical significance of Venn diagrams overlap was determined by hypergeometric testing analysis using the R package phyper. Abbreviations: A, acidity; Cd, cadmium; HL, high light; HS, heat stress; PQ, paraquat.

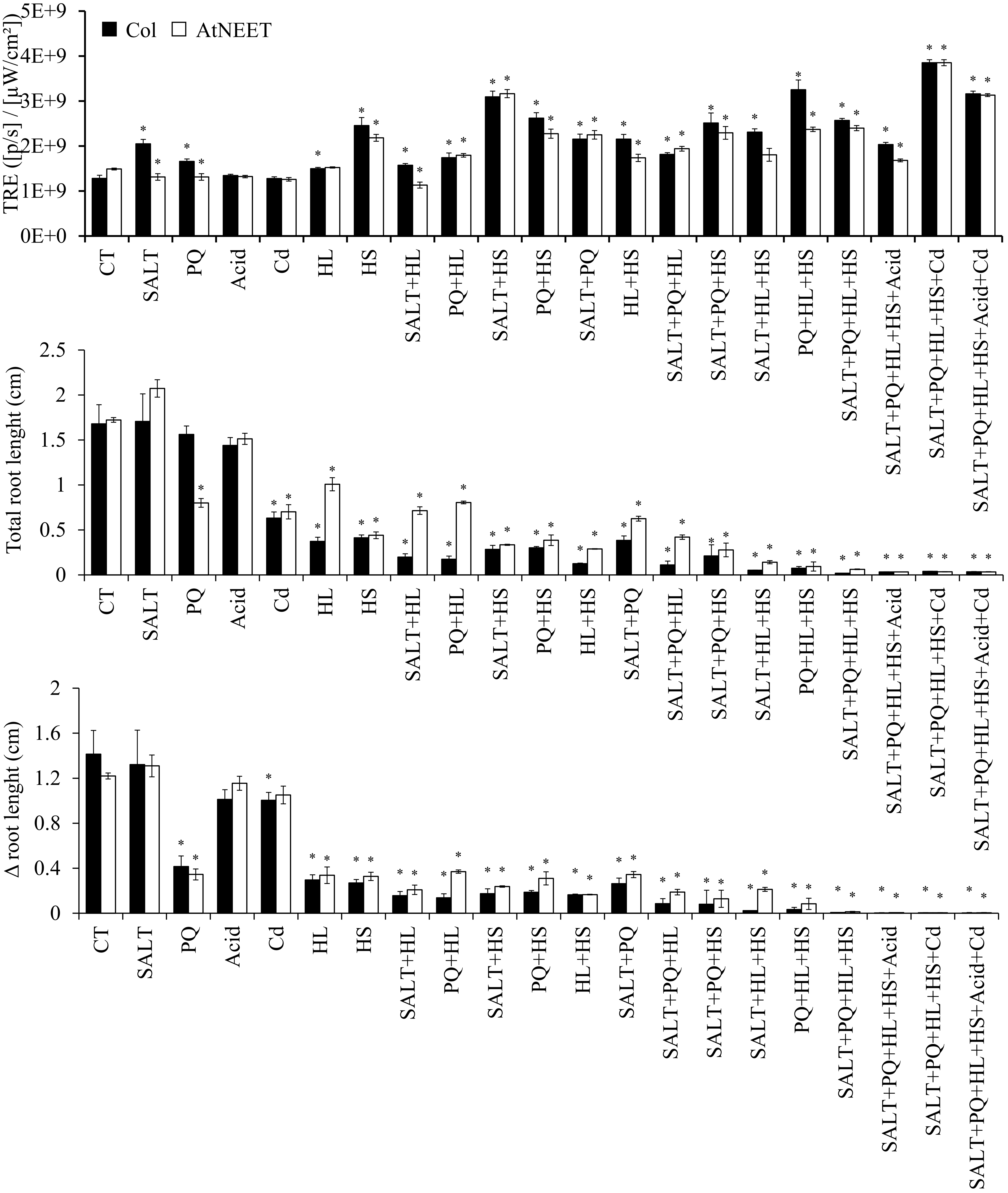

Fig. S6. Total and delta (Δ) root growth, and whole-plant ROS accumulation of Arabidopsis wildtype and AtNEET seedlings subjected to multifactorial stress combinations of heat, salt, light and oxidative stresses applied in all possible combinations. Results are presented as the mean ± SD. Statistical analysis was performed by two-way ANOVA followed by a Tukey post hoc test (asterisks denote statistical significance at $p < 0.05$ with respect to controls). Abbreviations: CT, control; PQ, paraquat; HL, high light; HS, heat stress; TRE, Total Radiant Efficiency.

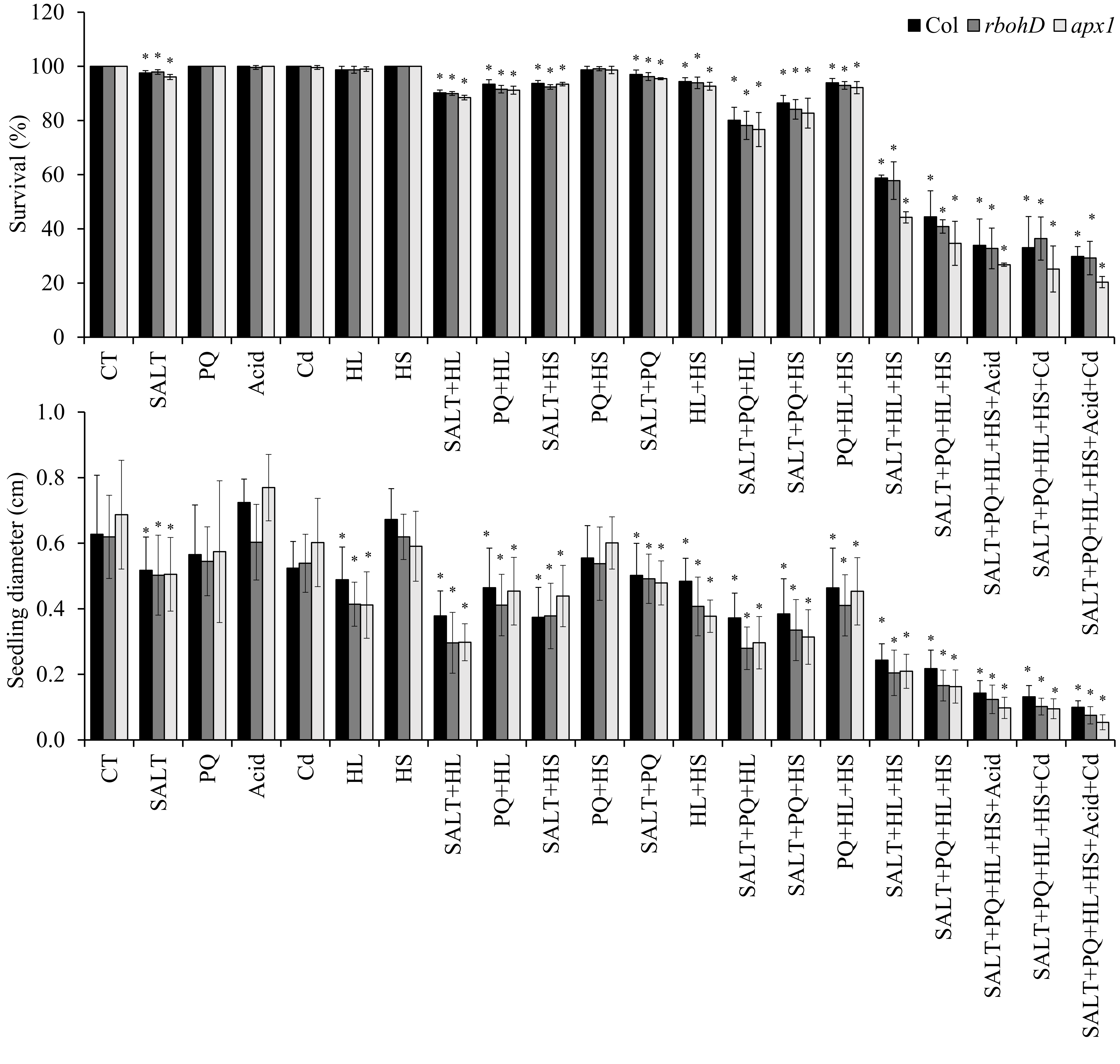

Fig. S7. Survival and seedling diameter of Arabidopsis wildtype, *rbohD* and *apx1* seedlings growing in soil subjected to multifactorial stress combinations of heat, salt, light, oxidative stresses, acidity and cadmium. Results are presented as the mean ± SD. Statistical analysis was performed by two-way ANOVA followed by a Tukey post hoc test (asterisks denote statistical significance at $p < 0.05$ with respect to controls). Abbreviations: Apx1, ascorbate peroxidase 1; RbohD, respiratory burst oxidase homolog D; CT, control; HL, high light; HS, heat stress; PQ, paraquat.

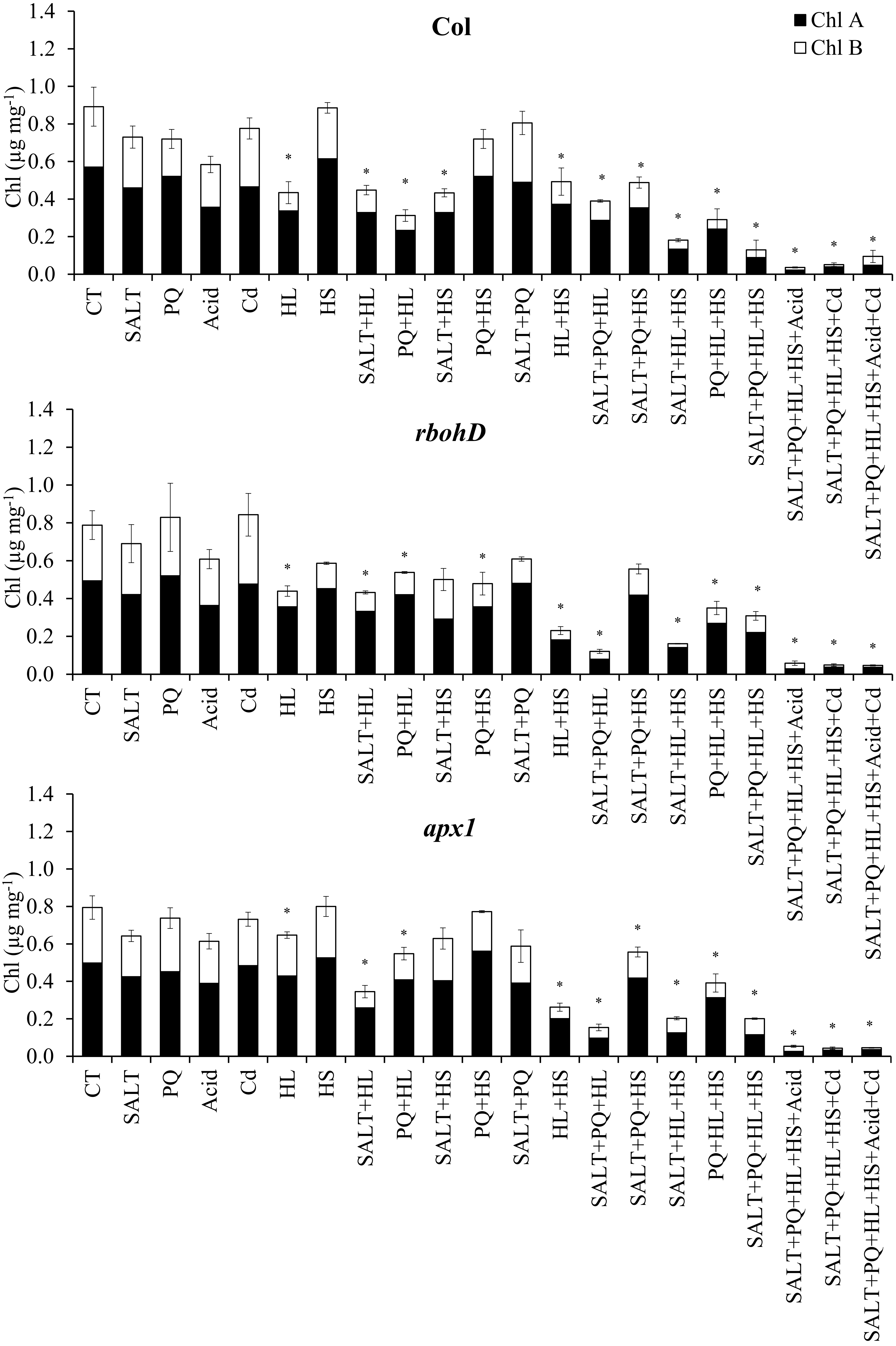

Fig. S8. Chlorophyll content of Arabidopsis wildtype, *rbohD* and *apx1* seedlings growing in soil subjected to multifactorial stress combinations of heat, salt, light, oxidative stresses, acidity and cadmium. Results are presented as the mean ± SD. Statistical analysis was performed by two-way ANOVA followed by a Tukey post hoc test (asterisks denote statistical significance at $p < 0.05$ with respect to controls). Abbreviations: Apx1, ascorbate peroxidase 1; RbohD, respiratory burst oxidase homolog D; Chl, chlorophyll; CT, control; HL, high light; HS, heat stress; PQ, paraquat.

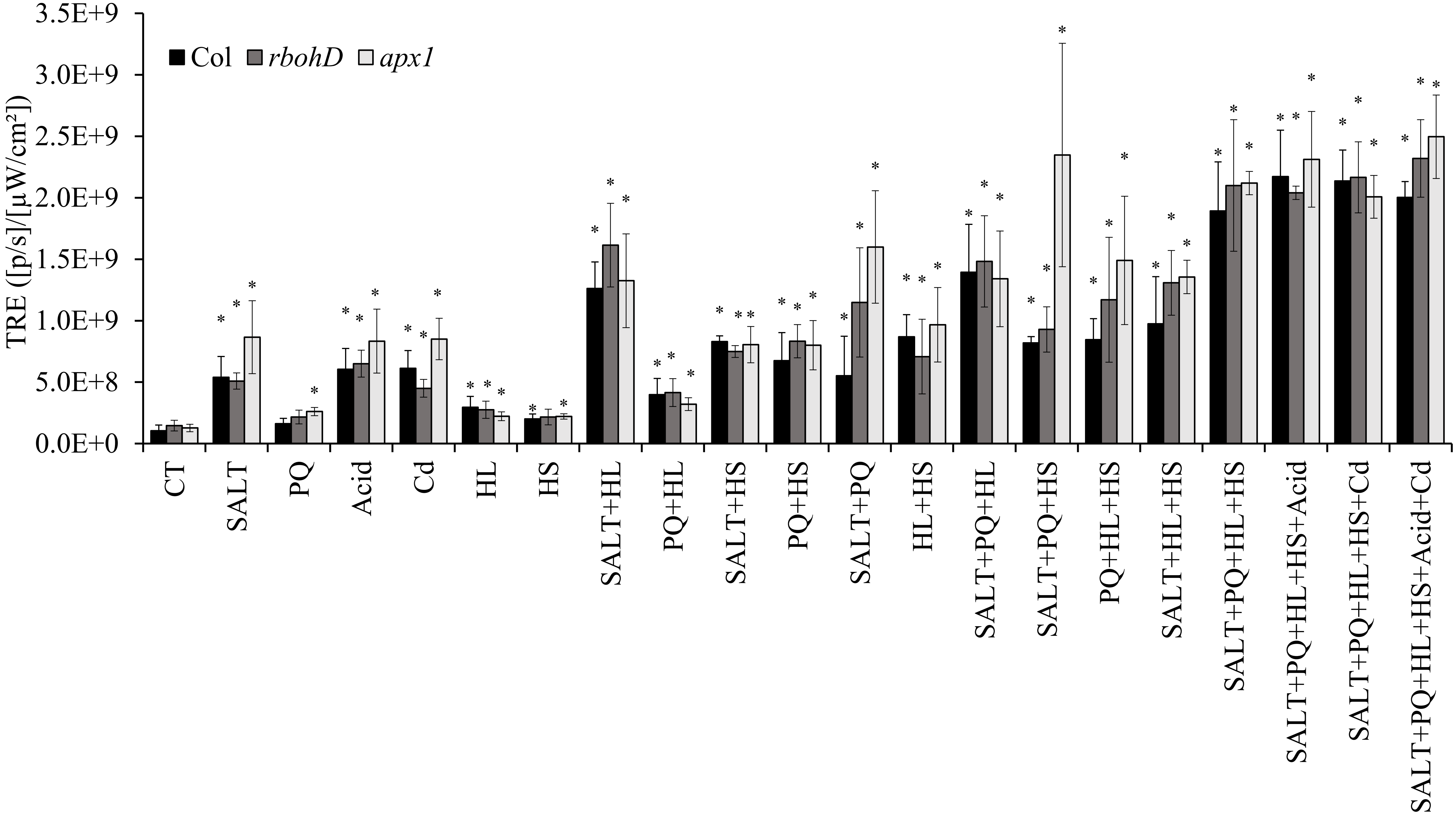

Fig. S9. Whole-plant ROS accumulation of Arabidopsis wildtype, *rbohD* and *apx1* seedlings growing in soil subjected to multifactorial stress combinations of heat, salt, light, oxidative stresses, acidity and cadmium. Results are presented as the mean ± SD. Statistical analysis was performed by two-way ANOVA followed by a Tukey post hoc test (asterisks denote statistical significance at $p < 0.05$ with respect to controls). Abbreviations: Apx1, ascorbate peroxidase 1; RbohD, respiratory burst oxidase homolog D; TRE, Total Radiant Efficiency; CT, control; PQ, paraquat; HL, high light; HS, heat stress.
